# Integrative analysis of next generation sequencing for small non-coding RNAs and transcriptional regulation in Myelodysplastic Syndromes

**DOI:** 10.1186/1755-8794-4-19

**Published:** 2011-02-23

**Authors:** Dominik Beck, Steve Ayers, Jianguo Wen, Miriam B Brandl, Tuan D Pham, Paul Webb, Chung-Che Chang, Xiaobo Zhou

**Affiliations:** 1 Bioengineering and Bioinformatics Program, The Methodist Hospital Research Institute, Weill Cornell Medical College, Houston, TX, 77030, USA; 2 School of Engineering and Information Technology, The University of New South Wales, Canberra, ACT, 2600, Australia; 3 Department of Pathology, The Methodist Hospital and The Methodist Hospital Research Institute, Weill Cornell Medical College, Houston, TX, 77030, USA; 4 Department for Genomic Medicine, The Methodist Hospital Research Institute and Department of Radiology, Weill Cornell Medical College, Houston, TX, 77030, USA

## Abstract

**Background:**

Myelodysplastic Syndromes (MDSS) are pre-leukemic disorders with increasing incident rates worldwide, but very limited treatment options. Little is known about small regulatory RNAs and how they contribute to pathogenesis, progression and transcriptome changes in MDS.

**Methods:**

Patients' primary marrow cells were screened for short RNAs (RNA-seq) using next generation sequencing. Exon arrays from the same cells were used to profile gene expression and additional measures on 98 patients obtained. Integrative bioinformatics algorithms were proposed, and pathway and ontology analysis performed.

**Results:**

In low-grade MDS, observations implied extensive post-transcriptional regulation via microRNAs (miRNA) and the recently discovered Piwi interacting RNAs (piRNA). Large expression differences were found for MDS-associated and novel miRNAs, including 48 sequences matching to miRNA star (miRNA*) motifs. The detected species were predicted to regulate disease stage specific molecular functions and pathways, including apoptosis and response to DNA damage. In high-grade MDS, results suggested extensive post-translation editing via transfer RNAs (tRNAs), providing a potential link for reduced apoptosis, a hallmark for this disease stage. Bioinformatics analysis confirmed important regulatory roles for MDS linked miRNAs and TFs, and strengthened the biological significance of miRNA*. The "RNA polymerase II promoters" were identified as the tightest controlled biological function. We suggest their control by a miRNA dominated feedback loop, which might be linked to the dramatically different miRNA amounts seen between low and high-grade MDS.

**Discussion:**

The presented results provide novel findings that build a basis of further investigations of diagnostic biomarkers, targeted therapies and studies on MDS pathogenesis.

## Background

Myelodysplastic Syndromes (MDS) are a group of heterogeneous hematopoietic stem cell disorders, which often lead to acute myeloid leukemia (AML). This group of diseases is most common in the growing demographic of the late sixties-early seventies [1]. In the United States the estimated number of new cases per year is about 40,000-76,000 with an attached cost of about 30.000 USD per person and year.

MDS is characterized by ineffective bone marrow hematopoiesis, leading to cytopenias [2], with a highly variable disease progression that ranges from a slow development over many years to a rapid progression to AML within a few months. Patients can be classified into risk groups, primarily based on bone marrow myeloblast counts [3,4]. These include refractory anemia (RA), describing an early disease stage (low-grade MDS) and the refractory anemias with excess of blasts (RAEB1, RAEB2), which represent the later stages of the disease (high-grade MDS). While the median survival times are relatively long in the low and intermediate-1 classes, 97 and 63 months respectively, they are considerably shorter in the later classes with 26 for the intermediate-2 and only 11 months in the high risk group [5]. Current treatment options are rare and show only limited success. They mainly include allogeneic stem cell transplantation, treatment with hypomethylating agents and Lenalidomide.

There is increasing evidence that dysregulation of a number of different molecular pathways is involved from the disease onset, however, clearly defined mechanisms remain elusive [6]. The accumulation of cellular death is a common trait for the early stage of MDS [7,8]. It is thought to counteract the proliferation of dysfunctional cells and is the key characteristic of ineffective hematopoiesis and marrow failure [9,10]. With the continued expansion of diseased cells, genetic damage accumulates and contributes to disease progression, which may result in the transformation to AML. The later stages of MDS have been implicated with angiogenesis and reduced apoptosis [11-15].

Recent studies have suggested that small non-coding RNAs (sRNAs), in particular microRNAs (miRNAs), contribute to the pathogenesis and progression of MDS [16,17]. However, very limited information on sRNA expression has been reported for MDS to date. To overcome this bottleneck, we performed high-throughput next generation sequencing of small RNAs (RNA-seq) in primary marrow cells of low- and high-grade MDS patients, together with matched controls. The relatively new technology of RNA-seq [18] is the method of choice for sensitive global detection of different sRNAs across an unparalleled dynamic range, and we detected sRNAs with read counts from ten to one million reads. The data obtained here suggest important roles for Piwi-interacting RNAs (piRNA), transfer RNAs (tRNA) and miRNAs, including many known and novel microRNAs star (miRNA*). Further functional analysis of miRNA/miRNA* showed that these species regulate disease stage-specific molecular functions and pathways, in particular, those known to be deregulated at the gene expression level. In addition, integrative bioinformatics modeling of our experimental data and bioinformatics databases identified the disease stage-specific regulation of the polymerase II promoter by miRNAs and transcription factors (TFs). This suggested a feedback loop that might contribute to the attenuation of miRNA expression in high-grade MDS.

## Methods

### Patient samples

Samples were obtained from patients presenting at The Methodist Hospital. The use of marrow samples was approved by The Methodist Hospital Institutional Review Board. All research described conformed to the Helsinki Declaration.

### High throughput small RNA sequencing and data analysis

RNA in the 18-30 bp range was isolated from a 15 percent urea-PAGE gel, and ligated to Solexa SRA5' and SRA 3' adapters, according to the standard protocol (available: http://www.illumina.com). Briefly, the SRA5' adapter was ligated to the 5' end of the selected RNAs. The ligation products were gel purified and SRA3' adapters ligated to their 3' ends. The resulting products were also gel purified, reverse transcribed and amplified with primers containing sequences complementary to the SRA5' and SRA3' adapters, after which they were gel purified again. The size and quality of the resulting libraries were verified using an Agilent DNA1000 Bioanalyzer chip (Agilent) and sequenced on a Solexa GAIIx, using PhiX as a loading control and analyzed with the standard Illumina Pipeline version 1.4. This produced approximately 13 million reads per lane.

In our analysis we used the s_x_sequence.txt files, containing 64 bit quality-scored output per-lane. The first 20bases of these reads were parsed in Mysql database tables, and further analyses utilized the MySQL database engine.

At this stage, the database was employed to identify and count distinct reads and to export this information into fasta formatted output files (Additional files 1, 2, 3). The results were used to map each small RNA to its matching position in the human genome. A variety of algorithms exists to perform this task including ELAND, which is provided with the Solexa GAIIx. However, a particular fast and memory efficient algorithm that outperforms other approaches is Bowtie [19]. This algorithm allows filtering alignments based on mismatches and can omit reads matched to multiple positions on the reference. The human genome version GRCh37 was downloaded from the NCBI website and converted into a bowtie index file. All distinct reads were aligned to this reference sequence. We allowed for at most two mismatches and only considered reads that aligned to at most 25 positions in the genome (parameter setting v = 2 and m = 25). With this parameter set, on average, 70 percent of the short sequence reads from all three lanes had positive matches to genome coordinates, about 21 percent did not match any genome position and about 10 percent had more than 25 matches.

A number of different databases were used as annotation basis for the aligned next generation sequencing reads. Information on sequences and genome positions of miRNAs were obtained from miRBase version 14. However, since our sample preparation and sequencing protocol is not specific for miRNAs, we downloaded information on other small RNAs from the UCSC genome browser. This contains genome positions for different small RNAs, including but not limited to tRNAs, rRNAs, scRNAs, suRNAs and srpRNA in the repeatmasker track, as well as positions of known exons. The sequences of known human piRNAs were searched and downloaded from the NCBI http://www.ncbi.nlm.nih.gov.

**Figure 1:**
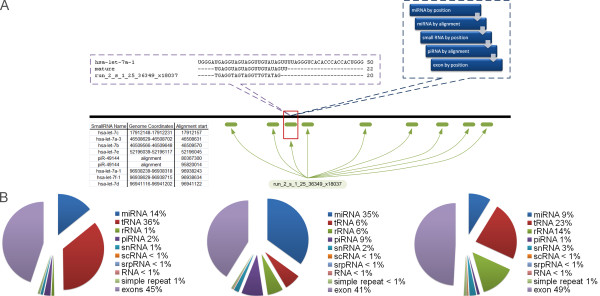
**NGS data analysis pipeline and comparison of sRNA annotations in MDS**. NGS data analysis pipeline used for this study. In A) we show the annotation of a sequence read. It was detected about 18000 times in RAEB2 and aligned at nine different positions, spread over six chromosomes, on the human genome (green). A single alignment position is shown (red) with the used annotation hierarchy (blue). The purple callbox, details the matched loci for miRNA let-7a-1, its full primary sequence (top), its mature sequence (middle) and the aligned short read (bottom). The brown callbox shows all nine annotations, including a number of miRNAs from the has-let-7 family as well as a piRNA. In B) we compare the total RNA content measured from our high-throughput sequencing and annotation steps, on the left results for the RAEB2, in the middle results for RA and on the right results for control.

The implemented annotation algorithm first checked if a read falls into a known miRNA loci (compare Figure 1). Unmatched reads were further aligned to primary miRNA sequences and perfect matches registered. If no match was identified, known loci for other small RNAs were searched in the following order rRNA, scRNA, sRNA, srpRNA, simple repeat and other RNAs. If a read was still uncharacterized, it was aligned against all piRNA sequences and matches returned for perfect alignments. Finally, if none of the above criteria was satisfied, positions for all human exons were first checked, if no match was identified reads were classified as unknown. The number of sequenced reads that annotated with a known RNA locus were used to represent its expression.

The read counts for miRNA and miRNA* were compared for the RA, RAEB2 and controls and significant differential expression defined following the example in [20]. We required that the ratio R of read counts in two different cells was within *R*_1 _> 1.5 ∨ *R*_2 _< 0.67 and the read count difference D within *D*_1 _> 100 ∨ *D*_2 _< -100. Consequently, over expression was defined by*R*_1 _and *D*_1 _and under expression by*R*_2 _and *D*_2_.

### Exon array profiling and data analysis

A total of 50ng RNA was extracted from each analyzed sample. We used primer provided from NuGEN and followed the manufacturer's protocol for the first strand cDNA synthesis. For RNA primer annealing, their mixtures were incubated for 2 minutes at 65°C and cooled to 4°C. After cooling, cDNA synthesis cycle followed; 4°C for 1 minute, 25°C for 10 minutes, 42°C for 10 minutes, 70°C for 15 minutes, and again 4°C for 1 minute. The second stranded reaction followed immediately. After mixing the first strand solution with second strand cDNA synthesis reaction solution, the entire mixture was incubated in the thermocycler as follows: 4°C for 1 minute, 25°C for 10 minutes, 50°C for 30 minutes, 70°C for 5 minutes, 4°C. Then, using the Agencourt^® ^RNAClean^® ^beads, the entire cDNA was purified according to the manufacturer's protocol. For the sense transcript cDNA generation, WT-Ovation™ Exon Module (NuGEN) was used. Based on the instructions in the manufacturer's manual, 3 μg of each cDNA was mixed with the provided primers and incubated for 5 minutes at 95°C and cooled to 4°C. After mixing with enzyme solution, the entire reaction mixture was incubated as follows: 1 minute at 4°C, 10 minutes at 30°C, 60 minutes at 42°C, 10 minutes at 75°C, and cooled to 4°C. Then the ST-cDNA was purified with the QIAGEN DNA clearing kit. After the purification, fragmentation reaction was carried out using FL-Ovation™ cDNA Biotin Module V.2 according to the recommended methods. Briefly, 5 μg of cDNA was mixed with the provided enzyme mix and incubated 30 minutes at 37°C and 2 minutes at 95°C. Then the reaction was cooled to 4°C. Next, the reaction was subjected to the labeling reaction as suggested by the manufacturer. The fragmented cDNA was mixed with labeling reaction mix and incubated at 37°C for 60 minutes and 70°C for 10 minutes. Then, the reaction was cooled to 4°C and used immediately for array hybridization. For the array hybridization, instead of recommended by Affimatrix, we used the standard array protocol provided by the NuGEN exon module. For hybridization, Chips were incubated in Gene Chip Hybridization Oven 640 and underwent the washing and staining processes according to the FS450_0001 fluidic protocol. Then, the array was scanned using Gene Chip Scanner 3000 (GCS3000).

The exon arrays for control, RA and RAEB2 were loaded into the Partek Genomics Suite 6.5. The Robust Multi-array Analysis (RMA) algorithm was used for initial intensity analysis [21] (Additional file 4). We generated gene expression estimates by averaging the intensities of all exons in a gene. Differential expression was defined as discussed for the NGS analysis above.

### Integrated target genes for MDS

In an earlier study Pellegatii and colleagues [22] used an Affymetrics Human Genome U133 Plus 2.0 GeneChip to assay consistently differentially expressed genes in hematopoietic stem cells (HSC) of 183 patients compared to 17 HSC of normal controls. This identified 534 probesets for RA and 4670 from RAEB2 patients. We matched these probesets to gene symbols and identified their corresponding transcript IDs on the Exon GeneChip. For the RA gene list, 69 probesets did not have annotated gene symbols, 103 had no corresponding transcripts and for 431 matching IDs were found. For the RAEB2 gene list, 807 probesets had no annotation, 1009 had no matching transcripts and for 3661 matching IDs were found. Altogether, this created a target gene space of 4092 probesets that were further analyzed by our bioinformatics modeling approach.

### Secondary structure and location of novel miRNA* sequences

The secondary structures for all miRNAs with stem-loop sequences deposited in miRBase were calculated using the Matlab Bioinformatics toolbox (version R2009a). The locations of mature miRNAs were identified as perfect alignments between the stem-loop and mature miRNA sequence. We calculated the locations of novel miRNA* sequences based on the genome coordinates of aligned small RNA reads. We note that due to mismatches in the miRBase alignments, e.g. between the miRNA stem-loop and the human genome, some derivations between the small RNA sequencing reads and the deposited stem-loop sequences may exist. All information was visualized using the tool VARNA [23].

### Prediction of miRNA-mRNA and miRNA*-mRNA pairs

Information on miRNA target genes was obtained from two popular and publicly available miRNA target prediction databases. We retrieved flat files for all predicted human miRNA targets available in miRanda [24] and targets conserved over different mammalian species from targetscan [25]. In order to reduce the number of false positive predictions we considered only targets predicted by both algorithms, which resulted in about 110.000 miRNA-mRNA pairs.

In theory the majority of miRNA* are degraded in the cell. Therefore, we restricted our analysis to sequences with minimum read counts of 100. In each case, we define a 7-mer nucleotide sequences based on the small RNA read with the highest copy number throughout the control, low and high risk MDS samples. The nucleotides at positions two to eight were extracted and transformed into the RNA alphabet. The seed regions were checked for overlap with other known miRNA and miRNA* sequences and the targetscanS algorithm was used to predict miRNA*-mRNA pairs, if the seed sequence was previously unreported. In general, this algorithm performs target predictions based on perfect and conserved matches between the genes untranslated region (UTR) and the first six nucleotides of the seed sequence. It further requires that the seed region is followed either by the nucleotide A (known as a t1A anchor) or that the position eight of the alignment contains a perfect Watson-Crick pairing. On contrast, if the seed sequences matched with a previously reported miRNA or miRNA*, we used the target prediction strategy as reported above.

### Prediction of transcription factor target genes

The flat files FACTOR and GENE of the commercially available database TRANSFAC v2008_2 [26] were downloaded and parsed into a MySQL database. The FACTOR and GENE flat files contain information on transcription factor proteins and genes regulated by transcription factors, respectively. A total of 2362 regulating factors for the human species (Homo Sapiens) were extracted and 70 entries, that did not describe proteins, but other regulatory factors were omitted. A large fraction (about 77 percent) of the remaining 2292 transcription factor proteins were mapped to Uniprot [27], either by external database ID's, or exact matches between protein names. With these accessions the protein coding gene IDs, as well as other information was downloaded automatically via a MATLAB based data retrieval algorithm implemented for this study. The transcript and probeset annotation files for the Affymetrix GeneChip Human Exon 1.0 ST Array were downloaded from the manufacture's website http://www.affymetrix.com and parsed into MySQL tables. Transcript IDs for 98 percent of the human transcription factor coding genes were extracted based on direct matches between gene names.

Genes that can potentially be up regulated when the transcription factor protein binds to a specific site in its promoter region are called transcription factor target genes. We extracted all target genes for human transcription factor proteins by joining a number of database tables. This revealed 3296 gene targets for the 2292 transcription factor proteins. We used direct matches between the target gene names, as well as additional entries, to identify corresponding transcripts on the Affymetrix GeneChip. This resulted in matches for 83 percent of the target genes.

### Functional analysis for miRNA and miRNA* targets

**Figure 2:**
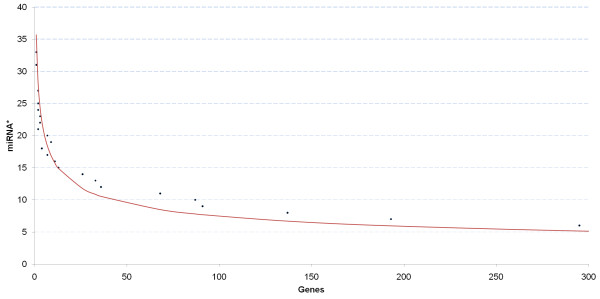
Threshold for miRNA/miRNA* target gene selection. This figure describes the number of genes (x-axis) that are targeted by different miRNA*s (y-axis), for the example of RA cells. In this particular case, we selected the threshold T to be 13 miRNAs and 93 different genes were selected for functional analysis.

The functional analysis of miRNA and miRNA* were performed by means of their predicted target genes. However, since the pools of potential target genes are large and suffer from high false positive rates, we selected only a limited set of genes for functional analysis. Therefore, we defined a threshold T describing the number of different miRNA or miRNA* that regulate a gene. Similar to many biological phenomena such functions are described by power laws (see Figure 2) and we aimed to select T in the exponential part of the function. This ensured that the selected genes were targeted by a large number of different miRNAs. We further tried to select at most 100 genes for the analysis. In each case, the selected target genes were imported into Ingenuity Pathway Analysis (IPA) version 8.5 and analyzed using the IPA Core Analysis algorithm.

### Data integration model and detection of important gene regulators

The proposed data integration model assumed that the mRNA amount present in a cell at any given time is linearly depended on the concentration of transcriptional acting TFs and post-transcriptional acting miRNAs. Therefore, gene expression was modeled as a linear combination of these factors plus random noise, which can be expressed following a standard regression model [28]

(1) $$y^{i}=\beta_{0}+\sum_{p=1}^{N}\beta_{p}x_{p}^{i}+\varepsilon$$

where *y^i ^*is the expression of gene *i*, *i *= 1,..., *G *with *G *being the number of genes under study, (*β*_0_,..., *β_N_*) are the regression coefficients to be estimated by our model, *N *sums up the number of TFs and miRNAs observed in the cells under study, *ε *is the noise term which is assumed an independent Gaussian random variable with expectation zero and variance *σ*^2^, $x_{p}^{i}$ was defined as

(2) $$x_{p}^{i}=\alpha_{p}^{i}\gamma_{p}\delta_{p}$$

where $x_{p}^{i}$ is a factor associating gene *i *with regulator *p*, *γ_p _*is a regulation characteristic and *δ_p _*the expression level of regulator *p*. The association $x_{p}^{i}$ was determined by miRNA and TF target prediction and $x_{p}^{i}$ was set to one if gene *i *was a target of regulator *p*, otherwise $x_{p}^{i}$ was set to zero. Transcription factors generally contribute to transcription and hence higher target genes levels, therefore, *γ_p _*was set to one if *p *was a TFs. On contrast, miRNAs are known to post-transcriptionally degrade mRNAs, hence *γ_p _*was set to minus one if *p *was a miRNA. The expression levels *δ_p _*were determined by experiments as discussed earlier. Note that all expression values were normalized to controls and standardized to mean zero and standard deviation one.

The above regression problem was solved using the recently proposed cyclical coordinate descent algorithm, which is based on an elastic net penalty [29]. This algorithm is particularly fast and the elastic net penalty is most appropriate to handle large and sparse problems (compare Additional file 5 Figure S1) of correlated inputs. In addition, it has the beneficial property of shrinking a number of predictor values *β_p _*to exactly zero, hence integrating an effective variable selection approach, otherwise computationally expensive [30]. Note, that the penalty is weighted and that these weights were determined by cross validation.

## Results and Discussion

### Defining the small RNAome of Myelodysplastic Syndromes by next generation sequencing

We performed high-throughput next generation sequencing of small RNAs (RNA-seq) on primary cells from control, low-grade (RA) and high-grade (RAEB2) MDS patients on an Illumina Genome Analyzer IIx (see Methods). This resulted in about thirteen million short sequence reads (length 38 bp) per sample. We implemented an annotation algorithm that integrates knowledge from diverse biological databases to characterize each RNA-seq read (Figure 1). In brief, all reads were trimmed (length 22 bp) and aligned against the current version of the human genome (GRCh37), using the publicly available software Bowtie [19]. We allowed for at most two mismatches between the reference and read sequences. Since, the analyzed reads were relatively short and we allowed mismatches, a large number aligned to multiple genome positions (green part Figure 1). Consistent with previous analyses, we decided to discard reads having more than 25 alignment positions [31]. For annotation, we matched small sequencing reads to a set of small RNAs that included miRNAs from miRBase [32], a number of other small RNAs, including tRNAs and rRNAs, from the RepeatMasker track of UCSCs genome browser [33], as well as piRNAs from the NCBI database http://www.ncbi.nlm.nih.gov (blue callout box Figure 1). This mapping showed that the composition of the small RNAome was dramatically different from the analyzed samples, suggesting a shift in the regulation of small RNA targets during the progression of this disease.

First, the relative amounts of tRNA to rRNA were significantly larger in RAEB2 compared to RA and control (36 vs. 1.6 and 1). Since tRNAs are vital building blocks for protein synthesis and required during translation, this may indicate an increased regulation of translation at this disease stage. A recent study based on tRNA microarrays reported a 20-fold elevation of tRNAs in tumor samples versus normal samples [34]. In addition, tRNAs have been shown to inhibit cytochorme c activated apoptosis [35,36]. Taken together, the high tRNA content may contribute to the two well known characteristics of high-grade MDSs, decreased apoptosis (in contrast to low-grade MDS) and high rate of leukemia transformation. To our knowledge, this novel finding has not been reported for MDS, highlighting the combined use of next generation sequencing and the proposed annotation methodology.

Next, the obtained sequencing data demonstrated the first evidence of piRNA expression in marrow cells, and particular enrichment in low-grade MDS. Piwi-interacting RNAs are a relative newly defined class of none coding RNAs with length from 26 to 32nt [37,38]. In RA their expression increased, accounting for about nine percent of total sRNA counts, compared to about two and one percent in RAEB2 and controls, respectively. The biogenesis of piRNA is not fully understood today, but increasing evidence pinpoints that PIWI proteins are required for the accumulation of piRNAs [39-42]. In accordance with this concept, our exon array data showed that *piwil1 *and *piwil2*, two of the four human PIWI coding genes, were significantly up-regulated in RA, compared to control and high-grade MDS cells. Furthermore, recent studies have indicated that the PIWI-piRNA complex may have a role in post-transcriptional silencing damaged DNA fragments [39,43,44] and that interrupting PIWI-piRNA formation can lead to DNA double strand breaks [45]. Altogether, these findings suggest that piRNA might be used as diagnostic markers for low-grade MDS, however, further studies of their role in MDS pathogenesis are warranted.

Finally, we found an increased regulatory role of miRNAs in cells of RA and RAEB2 patients. In low-grade MDS miRNAs represented about 35 percent of the total sRNAs, an almost 4-fold increase compared to control, highlighting their role in disease pathogenesis. Similarly, miRNA percentages were elevated to about 14 percent in RAEB2 compared to control, although at a lower extent (two-fold increase). Of note, miRNAs are currently the most widely studied species of sRNAs and they are known to influence mRNA levels as well as translation. Due to their profound effects, the above findings, and taken into account insufficient literature on miRNAs in MDS, we decided to further investigate and discuss their roles in MDS.

Sequencing of additional RNAomes is required to confirm the observed trends over a larger patient population.

### Detailed characterization of expressed miRNA loci and identification of novel miRNA*

**Figure 3:**
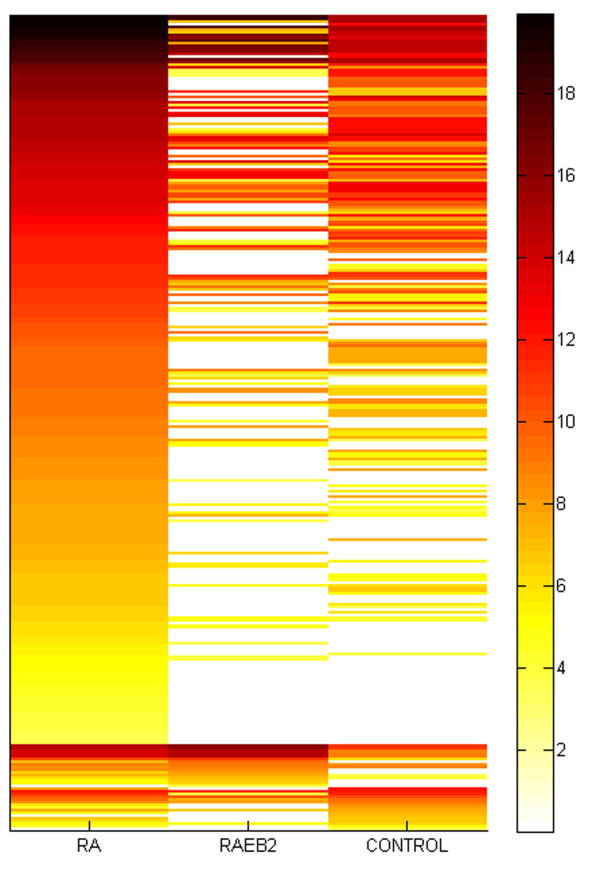
**Comparison of miRNA expression**. A heat map of the log2 transformed expression levels for miRNAs and miRNA* in the three analyzed samples.

In the analyzed samples, reads were found at 246 different full-length primary miRNA sequence loci. These included matches at 173 different mature miRNA sites in RA, 93 in controls and 79 in RAEB2. Expression varied between samples and was generally more elevated in RA compared to RAEB2 (compare Figure 3 and Additional file 6 Tables S1,S2 and S3). The miRNA hsa-mir-125b-2 was an exception and more elevated in RAEB2 (read counts: 264 RAEB2, 87 RA and zero in controls). A single miRNA, hsa-mir-720 (fold change 10), was significantly down-regulated in RA and no copies were detected in RAEB2. Furthermore, a total of 58 miRNAs were only expressed in RA (Additional file 6 Table S4), hsa-mir-191 was unique to controls and hsa-mir-9-3 was only detected in RAEB2.

**Figure 4:**
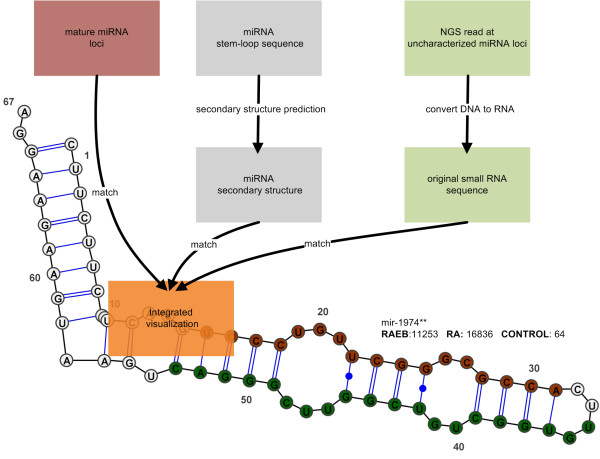
miRNA* analysis pipeline. Analysis pipeline for the visualization of novel miRNA* from small RNA sequencing reads aligned to uncharacterized loci on known primary miRNA sequences.

A number of high-throughput sequencing studies have recently reported the detection of miRNA*, often with higher copy numbers than their mature counterparts [46,47]. These studies further suggest that miRNA* associate with the effector complex AGO1 and regulate target gene expression. However, their roles in MDS have never been studied and we found reads matching to miRNA* motifs on 68 loci in RA, 55 in control and 24 in RAEB2 cells. In addition, multiple reads matched to uncharacterized positions on 59 different primary miRNA sequences. Interestingly, no miRNA* motifs had been reported for these loci before. Therefore, we visualized the secondary structure for their primary sequence, the location of the mature sequence and the reads clustered at uncharacterized loci (see Figure 4 Methods and Additional file 6 Table S5). Our bioinformatics analysis showed that most uncharacterized reads aligned on the miRNA* arm, opposite to the mature sequence. This has led to the definition of 59 previously unreported miRNA* candidates, of which 20 seed sequences have previously been associated in the targetscan database [48], but which did not exist in the miRBase version (v14) used for this study. We classified the remaining 39 motifs as novel miRNA* sequences (miRNA**) and folding information with locations on the miRNA arms are given in Additional file 6 Table S5.

Considering all samples together, significant expression was detected (read count at least 100) for 128 miRNA*, including 123 miRNA* in RA, 72 in control and 31 in RAEB2. Interestingly, in our RNA-seq data either the miRNA or the miRNA* (including miRNA**) arms were expressed at many miRNA loci (Additional file 5 Figure S2), suggesting a non-random and selective expression of the two different miRNA arms. Importantly, we found that 24 miRNA* were only expressed in RA, hsa-mir-24-1* was unique to control (copy number: 119) and no miRNA* was uniquely expressed in RAEB2. These miRNA* can potentially be used as biomarkers to diagnose low-grade MDS, which has significant overlapping morphologic and clinical features with reactive cytopenias, and is consequently very difficult to diagnose. However, further validation in additional patients and with different methods is needed to confirm these findings. Details for the ten miRNA* with the greatest fold changes in RA are given in Table 1 further information can be found in Additional file 6 Tables S1 and S4.

**Table 1:** Differentially expressed miRNA* and their target genes.

ID	fold	pval	miRNA* target genes (regulation)
mir-374b*	1613	5.44E-01	*HMGN2(↓),ZNF362(↑),LRRC8D(↓),NAV1(↑),ENAH(↓),SERBP1(↑),ENSA(***↑***),CDC42BPA(↑),SOCS5(↑),ANXA4(↓),CNNM4(↑),TLK1(↓),TFRC(***↑***),SMAD1(↑),PPARGC1A(***↑***),RCHY1(***↓***),HNRNPD(***↓***),PAM(↑),PURA(↑),RNF138(↑),YIPF5(***↓***),ANKRD6(↑),SCML4(↑),FAM160B2(↑),IKBKB(↑),PLAG1(***↑***),C9orf3(↓),ZFAND5(***↓***),SCD(↑),PTPRE(***↓***),SH3PXD2A(***↑***),SYT9(↑),RCC2(↓),OSBPL5(↑),CALCA(↑),HNRNPA1(***↓***),SIRT4(↑),HECTD1(***↓***),TRIM9(↑),TRMT5(↓),DUT(↑),LRRK1(↑),C15orf38(↓),ZFHX3(↑),FAM64A(↓),SMAD2(↑),SMAD7(↓),ZNF516(↓),MACROD2(***↑***),STX16(↓),ZBTB46(↑),RUNX1(↓)*
mir-374a*	1583	1.52E-02	*ZBTB40(↑),PTGER3(↑),NEGR1(***↑***),PCYOX1(↓),RANBP2(***↓***),DUSP19(***↑***),MGAT4A(***↓***),TFPI(↑),TNS1(↑),ZNF662(↑),PHLDB2(***↑***),EAF2(***↓***),DCK(↑),BMPR1B(↑),CAMK2D(↑),MTRR(***↓***),AFF4(***↓***),PHACTR2(↑),TULP4(↑),RNGTT(↓),KPNB1(↑),DPY19L4(↑),CCNE2(↑),CDC42(↑),PPP1R16A(↑),DLC1(↑),MTUS1(↑),ANKRD46(↑),TRPS1(↓),SAMD12(↑),ATAD2(↓),NFIB(↑),CUGBP2(***↓***),BCCIP(↑),SH3PXD2A(***↑***),RAG1(↑),RNF141(***↓***),CTSC(***↓***),KDELC2(↑),PARP11(↓),FRYL(***↑***),LYRM5(↓),NFE2(***↑***),XPO4(***↓***),EXOC5(↓),AFF1(↑),NOVA1(↑),SPTLC2(***↓***),CTDSPL2(↓),CCPG1(***↓***),NFAT5(***↓***),SMPD3(↑),STAT3(***↓***),MEX3C(***↓***),VASP(↓),MAPRE1(***↓***),RBL1(↑),REPS2(↓),MED13L(***↑***)*
mir-126*	1253	4.40E-01	*PRDM16(↑),KIF1B(↓),CHD1L(↓),PBX1(↑),ATP1B1(***↑***),PPP1R12B(↑),SNX25(↑),RPS6KC1(***↑***),TBCE(↑),LDLRAD2(↑),TMEM200B(↑),ZMYM6(↑),PTGER3(↑),NEGR1(***↑***),SASS6(↓),CEPT1(↑),ARNT(↓),GATAD2B(***↓***),KIF14(↑),NCOA1(↓),PPM1B(***↓***),ZAK(↑),STAM2(↓),CALCRL(↑),ALS2CR4(↑),ARL4C(↑),PHLDB2(***↑***),EIF2A(↓),MME(***↓***),EIF4A2(***↓***),GMPPB(↑),TMEM33(***↓***),EREG(***↓***),CCDC13(***↑***),BBS12(↓),PPARGC1A(***↑***),BTC(↑),TGFBI(↑),NDFIP1(↓),REEP5(↓),FOXF1(***↑***),UTRN(↑),ICK(↑),SRrp35(↓),AHR(↓),TRIP6(↑),LUC7L2(***↓***),HOXA7(↑),TFEC(↑),CHCHD3(↑),PTPRN2(↑),XKR6(↑),PSD3(↑),PLAG1(***↑***),FABP4(***↑***),MMP16(↑),TRPS1(↓),FAM120A(↓),ZFAND5(***↓***),AKNA(↑),CUGBP2(***↓***),SCD(↑),TRIM8(↓),HIPK3(***↓***),AASDHPPT(↓),PHF21A(***↓***),APOLD1(↑),FRYL(***↑***),LYRM5(↓),GALNT4(↑),XPO4(***↓***),COMMD6(***↓***),POU4F1(↑),C14orf39(↑),TERF2(↓),HS3ST3B1(↑),MLLT6(↓),YPEL2(↓),PRKCA(↑),SEC14L1(↓),GJC1(***↑***),HOXB2(↓),HOXB5(↑),SOCS6(↓),KIAA0355(↑),MYT1(↑),JAG1(↑),C20orf12(↑),ERG(↓),ZNF74(↓),MGAT3(↓),ST13(↓),REPS2(↓),ARMCX3(***↓***),NLGN4X(↑),MED13L(***↑***)*
mir-106a*	1176	3.36E-02	*CAMTA1(***↑***),MIER1(***↓***),HIPK1(***↓***),ENAH(↓),LUZP1(↓),GATAD2B(***↓***),CDC42BPA(↑),EPC2(↓),ARL4C(↑),HEG1(***↑***),ZFYVE28(↑),SGTB(↓),PURA(↑),SH3TC2(↑),RANBP9(***↓***),ICK(↑),KPNB1(↑),UBE2W(↑),RBM12B(↑),COL5A1(↓),ANKS6(↑),YME1L1(***↓***),PTPRE(***↓***),SEPHS1(↓),PLEKHA7(↑),MBD6(↓),KRAS(↓),SLAIN1(↑),XPO4(***↓***),GNPTG(↓),GINS3(↓),SSH2(↓),C18orf1(↑),HNRNPM(↑),NFIX(↑),ZNF473(↑),RBM39(***↓***),C22orf13(↓),RAC2(***↓***)*
mir-10a*	1134	3.43E-01	*PRDM16(↑),PBX1(↑),DBT(↑),CNNM4(↑),ARPP21(↑),TAPT1(***↓***),CYFIP2(***↓***),VDAC1(***↓***),DBN1(↑),C7orf58(↑),C7orf31(↑),MAGI2(***↑***),UQCRB(↓),NFIB(↑),RAG1(↑),DENR(↓),KRAS(↓),ACOX1(↓),SMCHD1(***↓***),STX16(↓),REPS2(↓),XK(↑),COL4A5(↑)*
mir-598**	733	1.92E-01	*PHF21A(***↓***)*
mir-20b*	672	1.00E-01	*STX12(↓),LEPR(***↑***),MAN1A2(***↓***),ANP32E(↓),PREPL(↑),SPTBN1(↓),TOP2B(***↓***),TBL1XR1(↓),BBS12(↓),UBE2D2(↓),FBXL17(↑),RANBP9(***↓***),BACH2(↑),DMTF1(↓),INPP5F(↑),AMOTL1(↑),PAFAH1B2(↓),PIP4K2C(↓),PACS2(↑),HECTD1(***↓***),TRIM9(↑),CTDSPL2(↓),TBC1D2B(***↓***),GINS3(↓),DNAJA2(***↓***),ANKRD11(↓),CDC6(↑),TWSG1(↑),NUMBL(↓),RP5-1022P6.2(***↓***)*
mir-195*	557.6	2.50E-02	*PPP1R12B(↑),SERBP1(↑),NEGR1(***↑***),RSBN1(↓),ARNT(↓),CDC42BPA(↑),C1orf96(↑),CENPO(↑),BCL2L11(↓),RAB1A(***↓***),WDR33(↓),ACVR1(***↑***),TLK1(↓),PPP2R5C(↓),ATP2C1(↑),MME(***↓***),KCNMB2(↑),LRIG1(↑),SUCLG2(***↑***),RAB6B(↑),DGKG(↑),HIGD1A(***↑)***,C4orf29(↑),PPP1R14B(↓),UBE2B(***↓***),SEC24A(***↓***),PURA(↑),FYB(↓),SEMA6A(↓),VDAC1(***↓***),TUBB(↓),NCOA7(↑),ZNF323(***↑***),ICK(↑),AHR(↓),SEMA3C(↑),FAM133B(↑),EPHB4(↑),TFEC(↑),PAXIP1(↑),PLAG1(***↑***),PAG1(***↓***),TRPS1(↓),SLC31A1(↓),LINGO2(***↑***),AKNA(↑),HSPA5(***↓***),C10orf119(↑),AMOTL1(↑),AASDHPPT(↓),PHF21A(***↓***),MRE11A(↓),BTG1(***↓***),CUL4A(↑),GNG2(***↓***),DACT1(↑),MPP5(***↓***),CFL2(↓),C14orf39(↑),ATP10A(↑),CCPG1(***↓***),GJC1(***↑***),SFRS2(***↓***),YES1(↓),OAZ1(***↓***),SIN3B(↑),BTG3(***↑***),ZNF280B(↑),CSNK1E(***↓***),ZFX(***↓***),STAG2(***↓***),BCOR(↓),ODZ1(↓),MED13L(***↑***)*
mir-16-1*	533.7	4.53E-02	*SPEN(↓),KIAA0495(↑),COL24A1(↑),PTPN22(***↓***),CD34(↑),FAM84A(↓),SLC5A7(↑),BCL2L11(↓),ARL6IP6(↓),UBE2E3(↓),COQ10B(***↓***),ABI2(***↑***),RAB1A(***↓***),SPRED2(↑),SCN2A(↑),STK39(↓),ALS2CR4(↑),TNS1(↑),ARL4C(↑),CDV3(***↓***),KCNMB2(↑),ANKRD28(↓),CDC25A(↑),KPNA1(↓),TBL1XR1(↓),KLF3(↓),FRAS1(↑),PPARGC1A(***↑***),EBF1(↑),MAPK14(***↓***),ZNF323(***↑***),BACH2(↑),CBX3(↓),NOD1(↑),RNF133(***↑***),CCNE2(↑),PAG1(***↓***),TP53INP1(***↓***),UQCRB(↓),SAMD12(↑),PALM2(↑),FUBP3(***↑***),PTEN(↓),HSPA5(***↓***),CUGBP2(***↓***),SUV39H2(↑),DDX21(↓),SCD(↑),RASSF4(↑),PDE3B(↓),CUL5(↓),ETS1(↑),FRYL(***↑***),KLF5(***↓***),FOXO1(↓),WDR76(↑),CCPG1(***↓***),SMPD3(↑),USP6(↑),MLLT6(↓),HLF(↑),SEC14L1(↓),TOP2A(↑),C18orf1(↑),ZCCHC2(↑),SMAD2(↑),ZNF516(↓),OAZ1(***↓***),MYT1(↑),JAG1(↑),RUNX1(↓),ZFX(***↓***),ZFY(***↓***),RBM3(***↓***),STARD8(↓),ODZ1(↓),NLGN4Y(↑)*
mir-503**	453	4.84E-03	*DBT(↑),MCL1(***↓***),ARHGEF2(↓),NCOA1(↓),SOCS5(↑),WDR33(↓),HIGD1A(***↑***),DHX15(***↓***),SLC12A2(↑),FNIP1(***↓***),SH3TC2(↑),GPR85(↑),TACC1(***↓***),MMP16(↑),UBR5(***↓***),TRPS1(↓),ZFAND5(***↓***),SUV39H2(↑),KBTBD3(↑),SLC43A1(↑),BACE1(↑),SUOX(↑),MON2(***↓***),DYRK2(↑),FRYL(**↑**),SENP1(↓),MLL2(↓),PCDH9(↑),CCNK(***↓***),IQGAP1(***↓***),AKT1S1(↓),MACROD2(***↑***),RP5-1022P6.2(***↓***),ZNF512B(↓),HSPA13(***↓***), STAG2(***↓***),NLGN4X(↑)*

List of ten miRNA* (see Additional file 6 Table S4 for folding information) that were detected with the largest fold changes in control and low-grad cells. We show the fold change, p-value (measuring if the number of down regulated target genes is greater than expected by chance) and target genes with regulation (bold arrows mark significant and italic non-significant regulation). We assessed the significantly down regulated genes for functional enrichment and pathways. The top five enriched biological functions included RNA Post-Transcriptional Modification (pval:1.2E-04), Cellular Growth and Proliferation (pval:1.25E-04), Cell Death (pval:5.79E-04) and Cancer (pval:5.95E-04-). The top six enriched canonical pathways included IL-22 Signaling (pval:2.63E-04), p53 Signaling (pval: 8.32E-04), IL-15 Signaling (pval:2.95E-03), B Cell Receptor Signaling (pval:4.47E-03) and FLT3 Signaling in Hematopoietic Progenitor Cells (pval:4.68E-03).

### Functional roles of miRNA and miRNA* in Myelodysplastic Syndromes

In order to identify biological functions that might contribute to low-grade MDS, and can be modulated by the detected miRNA/miRNA*, we first identified target genes for 91 miRNA and 104 miRNA* that were highest expressed in RA, compared to RAEB2 and control marrow cells. The total number of uniquely regulated mRNAs was 7021 for miRNA* and 4665 for miRNA (see Methods). To select high confidence targets, each gene was further ranked according to the number of miRNAs or miRNA* that potentially control its expression or translation (see Methods). This was necessary to counteract the high false positive rates of in-silico miRNA target predictions, which for example do not consider tissue specificity. From this ranking two gene sets (Table 2), the first consisting of 74 genes controlled by 19 miRNAs and the second consisting of 93 genes regulated by at least 14 miRNA*, were selected to compare significantly enriched molecular and cellular functions (Methods). Interestingly, four out of the top five functions, with the smallest p-values, overlapped. These included "Cell Death", "Cellular Development", "Cell Cycle" and "Gene Expression" (Table 2). The high compatibility suggested that the detected miRNA* fulfill similar roles to their mature counterparts, providing further evidence of their selectivity and biological importance.

**Table 2:** Enriched biological processes of miRNA and miRNA* target genes.

biological processes (pval)	Cell Death (1.84E-06)	Cellular Development (3.93E-06)	Gene Expression (8.34E-06)	Cell Cycle (1.05E-04)	Cellular Function and Maintenance (1.05E-04)
involved genes	*ACVR2B,BACH2 *(includes *EG:6046*8)*, CCDC6, E2F3, EGR3, HMGA2, IGF1, IGF1R,IKZF2, IRS2, MECP2, MIB1, NLK, NOVA1*	*CCDC6, CHD7, CNOT6L, DYRK1A, E2F3, EGR3, ESRRG, FNDC3A, HMGA2, IGF1, IGF1R, IGF2BP1, IRS2, MECP2, MIB1, MLL2, NLK, ONECUT2*	*ACVR2B, ATXN1, BACH2 (includes EG:60468), BAZ2A, BRWD1, CEP350, E2F3, EGR3, ESRRG, HMGA2, IGF1, IGF2BP1, JARID2, KLF12, MECP2, NFAT5, NFIB, NLK, ONECUT2*	*E2F3, ESRRG, IGF1, IGF1R, IRS2, JARID2*	*CLCN5, EGR3, IGF1, IGF1R, IRS2*
selected genes (miRNA)	*ACVR2B (↑),ADAMTS6 (↑),ANKRD52 (↓),ARPP-19 (N/A),ATXN1 (↑),BACH2 (↑),BAZ2A (***↓***),BRWD1 (***↓***),CCDC6 (↑),CEP350 (***↓***),CHD7 (N/A),CLCN5 (↑),CNOT6L (***↑***),CPD (***↓***),CPEB2 (***↓***),CPEB3 (↑),CPEB4 (↓),CSNK1G1 (↑),DCBLD2 (***↑***),DYRK1A (***↓***),E2F3 (↓),EGR3 (↓),EIF2C1 (↑),ESRRG (***↑***),ETNK1 (↓),FIGN (***↑***),FNDC3A (***↓***),GLT8D3 (↓),HIC2 (↑),HMGA2 (↓),IGF1 (***↑***),IGF1R (↓),IGF2BP1 (↑),IKZF2 (↓),IRS2 (***↓***),ITGB8 (↑),JARID2 (↓),JHDM1 D (N/A),KLF12 (↑),LIN28 (↑),LIN28B (↑),MECP2 (***↓***),MIB1 (↑),MIER3 (↓),MLL2 (↓),NFAT5 (***↓***),NFIB (↑),NLK (↓),NOVA1 (↑),NRK (↑),ONECUT2 (↑),OTUD4 (↓),PALM2 (↑),PAPD5 (***↓***),PBX3 (↓),PGM2L1 (↑),PLAG1 (***↑***),PLAGL2 (***↓***),PTPRD (↑),PURB (↓),QKI (***↓***),RNF165 (↑),RNF38 (***↓***),RPS6KA3 (↓),SNX16 (↑),SOCS6 (↓),SP1 (***↓***),SRGAP3 (↑),TBL1XR1 (↓),TGFBR1 (***↓***),TMCC1 (↑),TNRC6B (↓),ZBTB34 (***↓***),ZFHX4 (↑)*				
**biological processes (pval)**	Gene Expression (2.02E-09)	Cell Cycle (3.12E-05)	RNA Post-Transcriptional Modification (3.14E-05)	Cell Death (3.43E-05)	Cellular Development (5.40E-05)
**involved genes**	*ACVR2B, BACH2 (includes EG:60468), BCL11B, BMPR2, CBL, CREBZF, CTDSP2, DDX6, ESRRG, FGF7, FOXN3, HIPK2, HLF (includes EG:3131), IGF1, KLF12, MAF, MECP2, MEF2 D, MTF1, NFAT5, NFIB, ONECUT2, PBX1, PURB, SMAD4, SOX11, SP1, TEAD1, THRB, TRPS1, ZNF148*	*CBL, DCX, ESRRG, FGF7, FOXN3, IGF1, IGF1R, RPS6KA3, SMAD4, SP1, THRB*	*CNOT6L, CUGBP2, MBNL1, NOVA1, SFRS1*	*ACVR2B, BACH2 (includes EG:60468), BCL11B, BMPR2, CBL, CREBZF, CTDSP2, DDX6, ESRRG, FGF7, FOXN3, HIPK2, HLF (includes EG:3131), IGF1, KLF12, MAF, MECP2, MEF2 D, MTF1, NFAT5, NFIB, ONECUT2, PBX1, PURB, SMAD4, SOX11, SP1, TEAD1, THRB, TRPS1, ZNF148*	*ACVR2B, BCL11A, BCL11B, BMPR2, CBL, CNOT6L, COL11A1, DCX, DYRK1A, ESRRG, FGF7, HIPK2, IGF1, IGF1R, KCNMA1, MAF, MARCKS (includes EG:4082), MBNL1, MECP2, MEF2 D, MLL2, NDST1, ONECUT2, PBX1, PLAG1, RC3H1, SMAD4, SP1, THRB, ZFX*
selected genes (miRNA*)	*AAK1 (↓),ACVR2B (↑),ADCY1 (↑),AFF2 (↓),ANKS1B (↑),ARHGEF12 (***↑***),BACH2 (↑),BCL11A (↑),BCL11B (↓),BMPR2 (↑),BNC2 (↑),BSN (***↑***),C1orf21 (↑),CBL (***↓***),CNOT6L (***↑***),COL11A1 (↑),CPEB2 (***↓***),CREBZF (↓),CTDSP2 (↓),CUGBP2 (***↓***),DCX (↑),DDX6 (***↓***),DYRK1A (***↓***),ENAH (↑),ESRRG (***↑***),FGF7 (↑),FLJ20309 (N/A),FOXJ3 (↓),FOXN3 (↓),GATAD2B (***↓***),HELZ (***↓***),HIPK2 (↓),HLF (↑),HNRNPU (↓),IGF1 (***↑***),IGF1R (↓),IKZF2 (↓),JHDM1 D (N/A),KCMF1 (***↓***),KCNMA1 (↑),KLF12 (↑),LPHN2 (↑),MAF (↑),MAPK1IP1L (***↓***),MARCKS (***↓***),MBNL1 (↓),MECP2 (***↓***),MEF2 D (↓),MEX3A (N/A),MLL2 (↓),MTF1 (N/A),MYT1L (↑),NAV1 (↑),NDST1 (↑),NFAT5 (***↓***),NFIB (↑),NOVA1 (↑),NUFIP2 (***↓***),ONECUT2 (↑),PBX1 (↑),PHF15 (↑),PLAG1 (**↑**),PTPRD (↑),PURB (↓),RC3H1 (N/A),RIC8B (↑),RPS6KA3 (↓),SAMD12 (↑),SERBP1 (↑),SFRS1 (***↓***),SHANK2 (↑),SLC5A3 (↓),SMAD4 (***↓***),SMG1 (↑),SOX11 (↑),SP1 (***↓***),SPOPL (***↓***),STXBP5L (N/A),TEAD1 (↑),THRB (↑),THSD7B (N/A),TMEM170B (N/A),TNRC6B (↓),TRPS1 (↓),UBL3 (***↓***),ZC3H12C (N/A),ZCCHC24 (↑),ZFAND5 (***↓***),ZFHX4 (↑),ZFX (***↓***),ZNF148 (↓),ZNF609 (N/A),tcag7.1228 (N/A)*,				

This tables gives an overview of the selected miRNA (top) and miRNA* (bottom) target genes, their regulation (bold is used for significant expression and italic for non-significant expression), the top five molecular functions of these genes as well as the genes involved in these functions.

To study the overall role of miRNA/miRNA* in RA and RAEB2 cells, their target genes were combined for further analysis. In RA, we included 94 genes regulated by at least 27, and in RAEB2 a total 83 genes targeted by at least three different miRNA/miRNA*. The difference in the required number of regulating miRNA/miRNA* were attributed to the higher number of differentially expressed miRNA in RA (compare Additional file 5 Figure S3).

Next, we identified significantly enriched molecular and cellular functions (Methods) and compared results with a recent large scale gene expression study of 183 MDS patients [22].

In both disease grades the selected genes were enriched for the molecular function of "Cell Death" (RA: 9.86E-06, RAEB2: 1.75E-04). This is in agreement with the above study, which identified apoptosis as the main deregulated process in low-grade MDS.

Again consistent with the cited study, miRNA/miRNA* targets selected in both MDS subtypes were enriched for "DNA Replication, Recombination, and Repair " (RA:1.12E-03, RAEB2: 6.67E-03).

In addition, cell cycle regulatory genes were among the indentified target genes for both, RA and RAEB2. In accordance with the study cited above, we found that the "G2/M phase" (RAEB2:1.55 E-3) and "DNA damage checkpoint" (RAEB2: 6.67E-3) were exclusively regulated in RAEB2. On contrast the "G1 phase" (6.17E-06) was exclusive to RA.

These findings showed that miRNA/miRNA* interfere with molecular functions and pathways known to be deregulated at the transcriptomic level, as reported in the cited gene expression study (some additional information is given in Additional file 7). In the following we proposed a bioinformatics modeling approach to further elucidate the effects of miRNA/miRNA* on the MDS transcriptome.

### Computational modeling of transcriptome regulation in Myelodysplastic Syndromes

**Figure 5:**
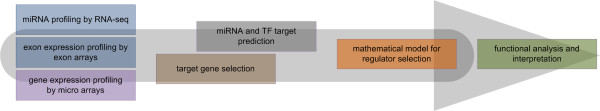
**Transcriptome analysis pipeline**. Pipeline for the integrative analysis of the MDS transcriptome, further described in the text and Additional file 5 Figure S4.

In the recent years it has become increasingly evident that miRNAs and TFs coordinate to regulate mRNA levels [49]. Consequently, we proposed a bioinformatics model that accounts for both effects. It integrated miRNA expression levels measured by next generation sequencing, gene expression measured by exons arrays, as well as data of a recently published gene expression microarray study [22]. All datasets were linked using a number of publicly and commercially available bioinformatics databases (Methods). In particular, we focused on the regulation of genes consistently differentially expressed over a large patient pool, that can be influenced by miRNAs/miRNAs* and TFs detected in our samples. The general workflow is illustrated in Figure 5 and we briefly describe the main aspects below (more information is given in the Methods section and Additional file 5 Figure S4).

The analysis started with miRNA profiling in samples of RA and RAEB2 patients by next generation sequencing, as discussed earlier.

In addition, we measured gene expression and splice form variations using the Affymetrix GeneChip Human Exon 1.0 ST Array. In an earlier study the bone marrow of 55 RA and 43 RAEB patients were compared against 17 controls and genes collectively differentially expressed explored [22]. These differentially expressed genes were merged with the exon array profiling (Additional file 5 Figure S5) and a set of 385 RA and 2795 RAEB2 genes was constructed.

Again, bioinformatics databases were used to map between the obtained gene lists and interacting miRNAs and TFs. This identified about 10.000 possible interactions between 217 miRNA (94 miRNA and 123 miRNA*), either expressed in RA or RAEB2, and their corresponding genes.

In a similar step all known human TF proteins and their validated promoter targets were identified. Next, their coding genes were determined using a retrieval algorithm which automatically queries the Universal Protein Resource [27]. The coding gene IDs were then mapped to Affymetrix transcript IDs to obtain gene expression levels from the analyzed exon array. After TFs with low expression levels were erased, 198 TFs with 465 validated interactions to the described MDS gene pool could be identified.

However, 1073 genes could not be associated with an expressed miRNA nor a TF, and thus potential secondary targets were omitted from further analysis.

The obtained expression levels for all miRNA/miRNA*, TF and genes were normalized to their respective controls and then standardized to a mean of zero and a standard deviation of one.

To develop a bioinformatics model for gene expression regulation, we assumed that the mRNA amount, present in a cell at any time, is linearly dependent on its positive acting TFs and negative acting miRNAs [50,51]. Hence, the mRNA amounts can be modeled as a linear combination of the standardized expression levels of miRNAs and TFs. Note that all expression measures for genes, miRNA and TF were acquired from marrow cells of the same patients, whereas the other mentioned studies relied on expression levels from multiple studies of different tissues.

The resulting model for RA consisted of 1640 equations to represent each RA gene and 415 predictors (regulators, e.g. miRNA and TFs). For RAEB2 we used 1216 equations and 290 predictors.

In spite of the huge variable space, we were interested to determine how much each regulator contributes to the expression of the analyzed genes. This is a particular large regression problem and our input data, similar to other biological measurements, was highly correlated. In addition, the average number of miRNA and TF regulators per gene was small compared to the variable space (see Additional file 5 Figure S6), leading to a set of sparse equations, which posed another algorithmic difficulty.

To overcome these issues, we applied the recently proposed elastic net algorithm [29] that is specifically equipped to handle large, correlated and sparse problems. In addition, its regularization term was designed to shrink a numbers of predictors to exactly zero. This eliminates variables (miRNAs and TFs) without importance, and directly incorporates a feature selection procedure, which is otherwise computationally expensive.

In RA this strategy identified 349 variables, out of 415, with coefficients different from zero. Similarly, for RAEB2 it selected 197 out of the 290 possible variables. In order to rule out the possibility that these results are purely dependent on the expression levels of the regulators, or the number of regulated genes, we calculated a series of correlation coefficients. With Pearson Correlation Coefficients of 0.003 and 0.067 for the expression and 0.062 and 0.007 for the number of regulated genes, there were no correlations found for the low- and high-grade MDS, respectively.

The selected variables for RA included 119 miRNA*, 90 miRNA and 140 TF. In addition to the increased expression of miRNA* in RA and their potential to regulate low-grade MDS associated biological functions and pathways, the large selection of miRNA* provides further mathematical evidence for their regulatory importance.

**Figure 6:**
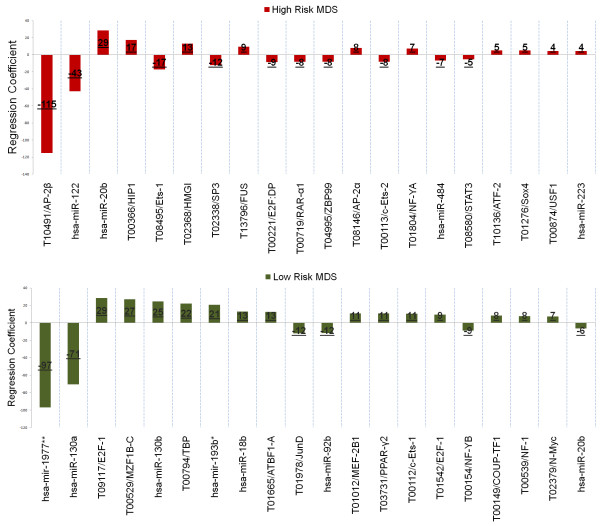
**MDS transcriptome regulators**. Top 20 regulators determined by the proposed modeling approach. The y-axis shows the regression coefficients and the x-axis lists the regulator names. We named TF with their transfac accession and the corresponding protein name. The miRNAs are named with their miRBase accession and we marked previously known miRNA* with a single and novel miRNA* with two stars. In addition, we indicate the rounded regression coefficients on the respective regulator bars.

To identify important miRNA/miRNA* and TFs, all regulators were ranked based on the aberration of their regression coefficients from zero (Figure 6). A large deviation, in positive or negative direction, is synonymous with a large influence on gene expression.

In RA, two subtype-specific expressed miRNAs were selected as most dominant regulators. Whereas the differentially expressed target genes of hsa-mir-1977** regulate hematopoiesis and apoptosis, hsa-miR-130a has previously been associated with the regulation of angiogenesis and platelet physiology [52,53]. The transcription factor E2F1 ranked three and is known to regulate S-phase dependent apoptosis in MDS [54,55]. Similar, eight out of 13 TF within the top 20 have previously been associated with "Hematological Disease" or "Hematopoiesis".

For RAEB2, the proposed pipeline selected 46 miRNA*, 76 miRNA and 84 TFs as influential. The 20 highest ranked regulators included 16 TFs, of which 12 have previously been associated with either "Hematological Disease" or "Hematopoeisis". The top ranked TF, AP-2β, has a known role in the development of metastatic phenotypes as well as apoptosis [56]. The highest ranked miRNAs were hsa-miR-122 and hsa-miR-20b, both expressed moderately and not linked to the RAEB2 phenotype.

In conclusion, the ranking of miRNAs and TFs with known and important relation to MDS shows the power of our approach. While a few TF have already been extensively investigated in MDS, an in-depth understanding of miRNA regulation remains elusive. We are planning to further study the functions of the novel miRNAs hsa-mir-1977** and hsa-miR-130a in primary cells to confirm our findings and illustrate their roles in MDS.

### Key functions regulated by miRNAs and TFs in Myelodysplastic Syndromes

**Figure 7:**
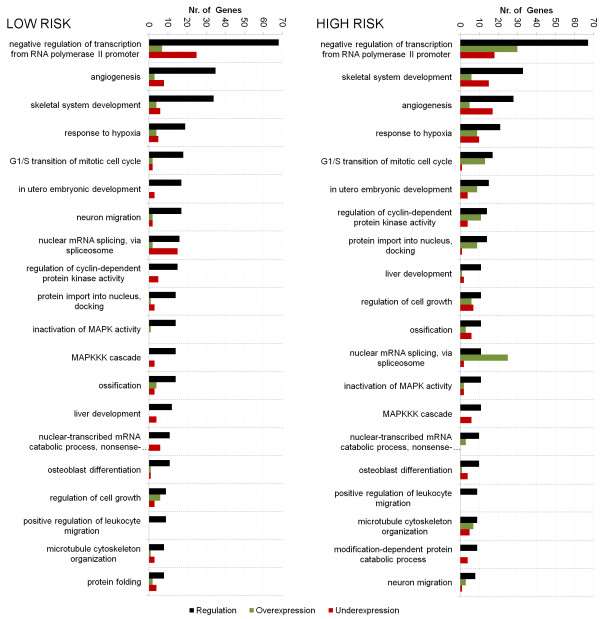
**MDS regulated biological processes**. Illustration of biological processes that are highly regulated by influential miRNAs and TFs, as selected by our in-silico model. The left figure shows results for the low risk and the right figure for the high risk grade. In both graphs the x-axis describes the regulated process. The y-axis shows, in the black bar, the number of selected miRNA and TF that regulate a certain processes. In the red bar the number of down- and in green bar the number of up regulated genes are shown.

In order to identify molecular processes influenced by the above regulators, we first annotated the target genes of highly ranked miRNAs/miRNA* and TFs (e.g. absolute regression coefficients greater than one) with pre-filtered (e.g. having less than 500 genes) gene ontologies [57]. Then each biological process was ranked according to the number of involved target genes. Further, genes differentially expressed in each process term were identified and overlaid with the above ranking onto Figure 7.

Some highly regulated processes, such as angiogenesis, were shared between low- and high-grade MDS. Moreover, our model indicated a few biological processes that are highly regulated in both disease subtypes, but different in the levels of their expression. For example "nuclear mRNA splicing, via spliceosome", "G1/S transition of mitotic cell cycle" or "protein import into the nucleus, docking ". Rationally, such processes are potential keys that can define functional differences in MDS subtypes.

Of particular interest was the process "negative regulation of transcription from RNA polymerase II promoters" (GO:0000122), which was the most regulated process in both MDS grades. This pathway prevents or reduces transcription of different RNAs, including miRNAs.

In RA, the majority of the differentially expressed genes in this term were down regulated (Figure 7), hence promoting transcription. By contrast in RAEB2, the majority of differentially expressed genes were up regulated, leading to a reduced RNA production.

Therefore, these results are in agreement with our earlier findings that some miRNAs were only detected, or had higher copy numbers, in RA compared to RAEB2.

Altogether, these results suggested that the differences in miRNA expression between RA and RAEB2, and potentially their downstream targets, might be the result of RNA polymerase II promoter regulation. In RA, this would indicate a potential feedback system in which expressed miRNA and TF down regulate "GO:0000122". In turn, this could increase expression of RNA and hence accumulate miRNAs. By contrast in RAEB2, the selected miRNA and TF up regulate "GO:0000122". This drives the cell to reduce RNAs synthesis and consequently decreases their overall amount.

Thus, the discussed feedback loops are a potential explanation for the high amounts of miRNA seen in RA and the much lower amount in RAEB2, two obvious discoveries from the RNA-seq analysis described above. Further studies to investigate the role of this pathway in MDS are warranted.

## Conclusions

In this paper we presented the first systematic profiling for small RNAs in Myelodysplastic Syndromes using next generation sequencing on the current Illumina Genome Analyzer IIx platform. A custom data analysis pipeline that handled raw reads, sequence alignment, data storage as well as integrative read annotation was implemented. The analysis showed that the small RNAome in low-grade MDS (RA) was enriched for piRNAs, potentially protecting DNA from the accumulation of mutations, a mechanism not observed in high-grade MDS (RAEB2). By contrast, tRNAs were enriched in RAEB2, which might contribute to the characteristic reduction in apoptotic cell death at this disease stage. In both grades a number of differentially expressed miRNAs and miRNA* were detected and 48 previously unreported miRNA* exposed. In all analyzed cells, miRNA reads were often found for either the mature or the star sequence, indicating selective expression of miRNA and miRNA*. Subsequent functional analysis of target genes showed that both miRNA species (i.e. miRNA and miRNA*), regulate similar MDS stage specific molecular functions and pathways indicating that miRNA* also play important regulatory roles on the MDS transcriptome. Using integrative bioinformatics modeling, we identified miRNA species and TFs that act as important regulators for a MDS transcriptome that is consistently deregulated over a large MDS patient pool. Further ontology analysis identified the geneontology process of "negative regulation of transcription from RNA polymerase II promoters" as highly controlled in both MDS grades. Additionally, our findings suggested a potential feedback loop, where specific miRNAs and TFs regulate their own expression by either enhancing polymerase II promoter function, as seen in RA, or repressing its function, as found in RAEB2. Further studies are warranted to experimentally substantiate our observation and to develop novel biomarkers for the diagnosis and treatment of MDS.

## Competing interests

The authors declare that they have no competing interests.

## Authors' contributions

XZ and CCC designed the study. SA performed the RNA-SEQ and JW the exon arrays. DB performed the data analysis, wrote the manuscript and contributed the study design. XZ and TP supervised the data analysis. CCC and PW supervised the data generation. MB contributed to the data interpretation and manuscript writing. All authors read, assisted with editing, and approved the final manuscript.

## Pre-publication history

The pre-publication history for this paper can be accessed here:

http://www.biomedcentral.com/1755-8794/4/19/prepub

## Supplementary Material

Additional file 1 **This file contains all unique sequence reads in fasta format for the control population**. The identifiers contain the number of times a read was sequenced, e.g. the x251 for the identifier run_2_s_5_25_1_x251 means the read was sequenced 251 times. Click here for file

Additional file 2 **This file contains all unique sequence reads in fasta format for the RA population**. The identifiers contain the number of times a read was sequenced, e.g. the x251 for the identifier run_2_s_5_25_1_x251 means the read was sequenced 251 times. Click here for file

Additional file 3 **This file contains all unique sequence reads in fasta format for the RAEB2 population**. The identifiers contain the number of times a read was sequenced, e.g. the x251 for the identifier run_2_s_5_25_1_x251 means the read was sequenced 251 times. Click here for file

Additional file 4 **This file contains the summarized gene expression levels as log intensities for the control, RA and RAEB2 populations**. Click here for file

Additional file 5 **This file contains the supplemental Figures referenced in this article**. Click here for file

Additional file 6 **This file contains the supplemental Tables referenced in this article**. Click here for file

Additional file 7 **This file contains the supplemental Text referenced in this article**. Click here for file
