# Supplementary material for: Integrative analysis of next generation sequencing for small non-coding RNAs and transcriptional regulation in Myelodysplastic Syndromes
Source: BMC Med Genomics. 2011 Feb 23;4:19. doi: 10.1186/1755-8794-4-19 (PMC3060843; doi:10.1186/1755-8794-4-19)
Supplement: Additional file 5 — This file contains the supplemental Figures referenced in this article. [file 1755-8794-4-19-S5.DOC]

**Supplementary Figure Legends**

**Figure S1.** This graph shows the regulation pattern for the analysed genes. The horizontal axis describes the genes (gene names not shown for readability) and the vertical axis the number of factors that regulate the gene. We visualized the regulation by a transcription factor in blue and positive values and the regulation by a miRNA in red and by negative numbers. It can clearly be seen that more regulating miRNA are known (or are predicted) when compare to transcription factors.

**Figure S2.** Distribution of small RNA sequencing reads into mature (red), star (green) and novel (orange) loci. The x-axis describes the miRNA loci, names are not shown for readability, and the y-axis is the log-transformed number seuqencing reads. At most miRNA loci, either the miRNA, the miRNA* or the novel sequence were selectively expressed. In other cases relatively more reads were found at mature compared to the miRNA* loci.

**Figure S3.** The venn diagram in A) shows total numbers of miRNA* with significant copy numbers when low (green) and high risk (red) cells are compared to controls. In total we found 128 miRNA* to be significantly expressed over all three cell populations. A total of 108, of these miRNA* are higher expressed in low risk and 12 in high risk MDS cells. Intrestingly 10 out of these 12 miRNA* are significant expressed in low and high risk MDS, altough eight out of these have higher copy numbers in low compare to high risk MDS. In total we found 175 miRNA expressed over all three cell populations. The venn diagram in B) shows the number of miRNA that have signifcant higher copy numbers in low (green) and high risk (red) cells compared to controls. Similar to the miRNA*, the majority of 48 miRNAs in the overlap between the two MDS grades was higher expressed in the low risk case. The Venn diagrams were visualized using the “Venn Diagram Plotter” tool availible at http://www.omics.pnl.gov.

**Figure S4.** Detailed outline of the bioinformatics modelling pipeline to measure the effects of different miRNA and miRNA*, as well as TF, on a fraction of the MDS transcriptome. This integrative approach combines gene expression and miRNA expression from our own experimentation with data available from a large scale expression profiling study and bioinformatics databases.

**Figure S5.** This figure shows the overlap in differentially expressed genes between the large scale gene expression study of bone marrow cells of 183 MDS patients (Pellagatti et al. 2010) and the exon array profiling of bone marrow cells of two MDS patients in our laboratory. The Venn diagram in A) shows down regulated genes in RA (green) and RAEB2 (red) and the Venn diagram in B) shows the up-regulated genes. The Venn diagrams were visualized using the “Venn Diagram Plotter” tool availible at http://www.omics.pnl.gov.

**Supplementary Figures**

| 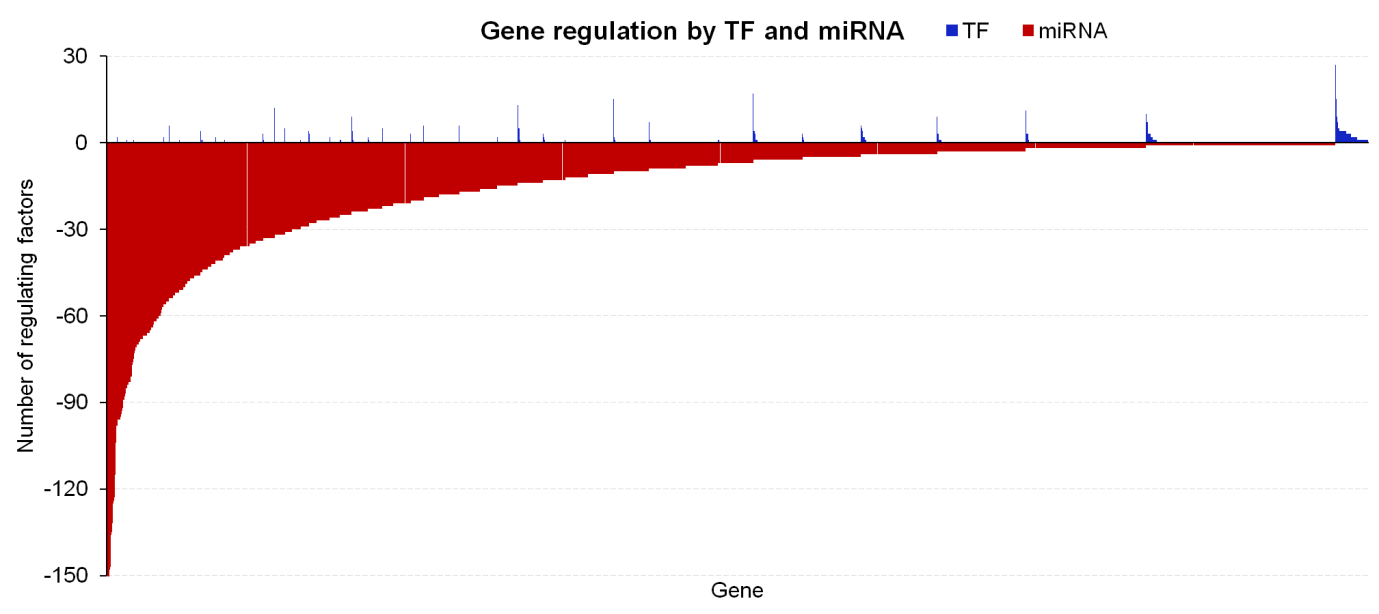 |
| --- |
| Figure S5. |

| 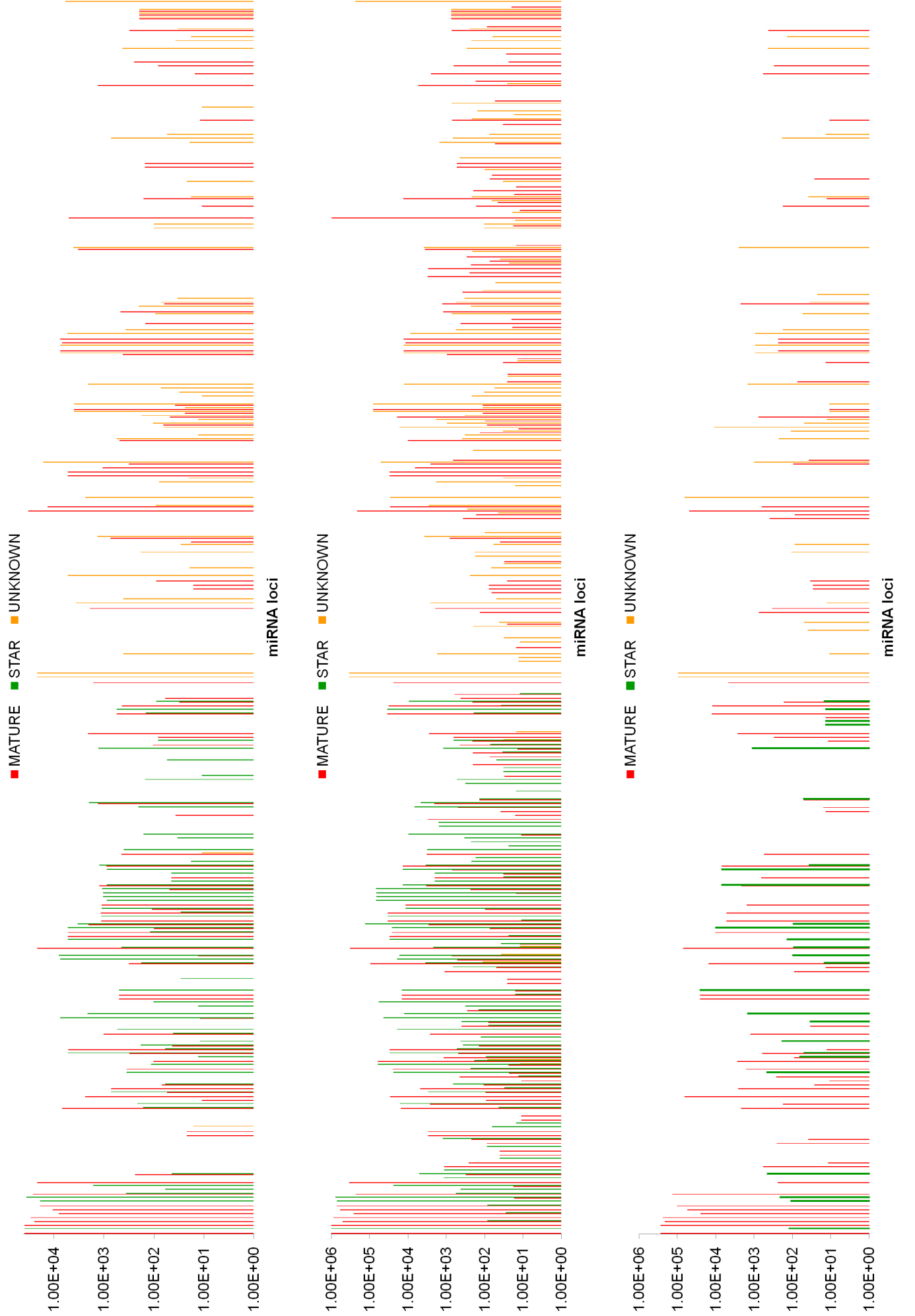 |
| --- |
| Figure S2. |

| 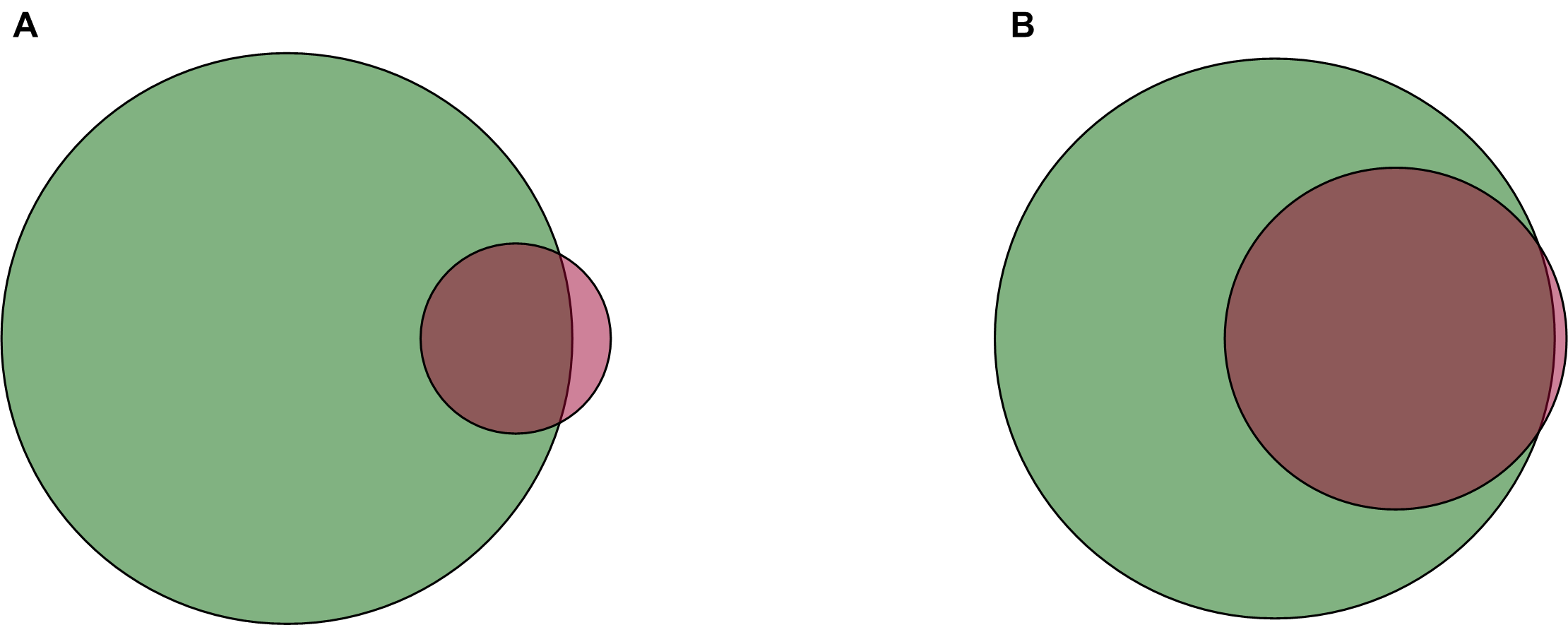 |
| --- |
| Figure S3. |

| 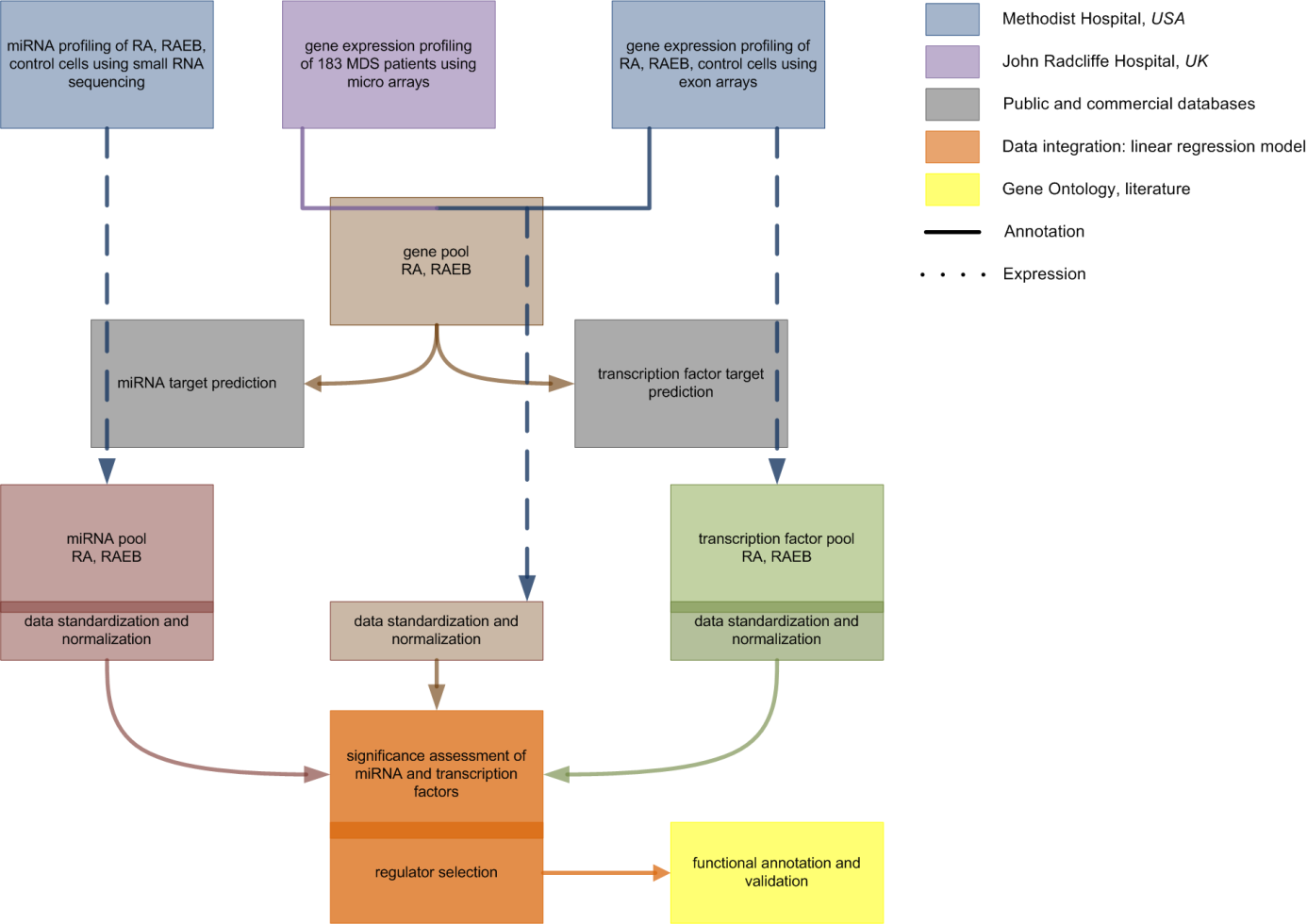 |
| --- |
| Figure S4. |

| **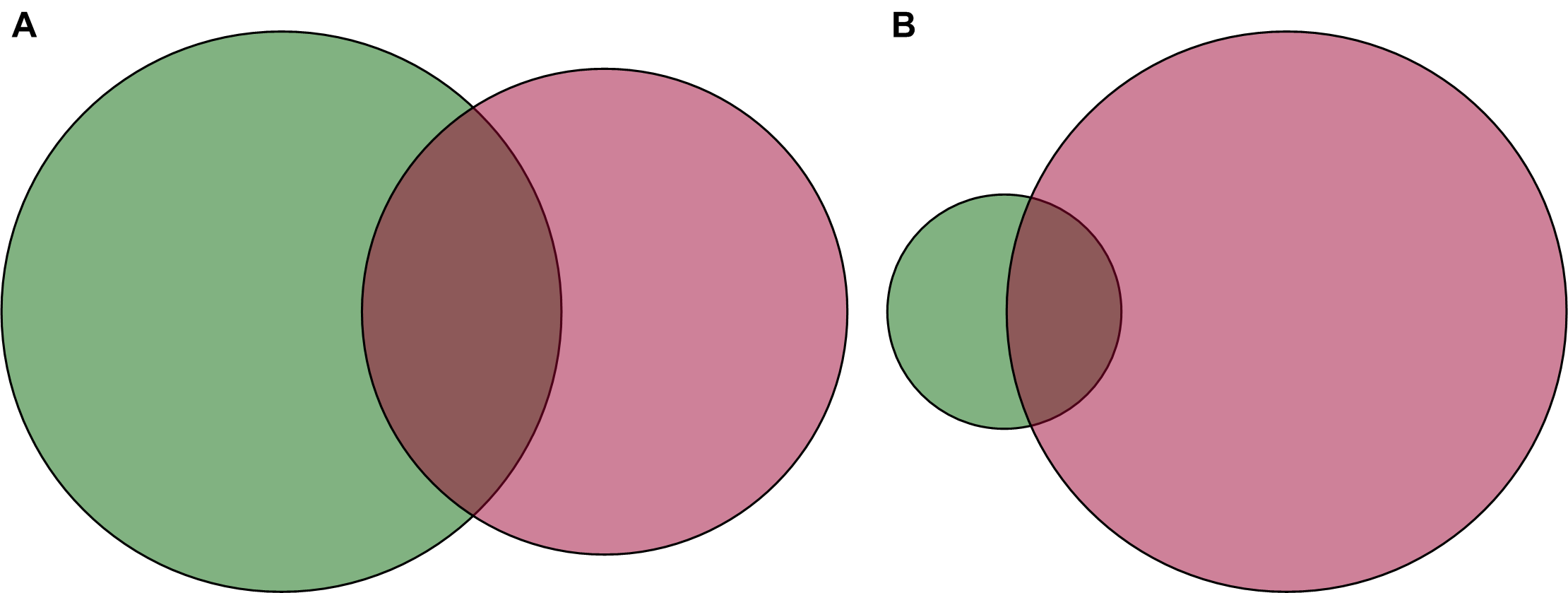** |
| --- |
| Figure S5. |

**References**

**Pellagatti A, Cazzola M, Giagounidis A, Perry J, Malcovati L, Della Porta MG, Jadersten M, Killick S, Verma A, Norbury CJ et al. 2010. Deregulated gene expression pathways in myelodysplastic syndrome hematopoietic stem cells. *Leukemia* 24(4): 756-764.**
