# Supplementary material for: Integrative analysis of next generation sequencing for small non-coding RNAs and transcriptional regulation in Myelodysplastic Syndromes
Source: BMC Med Genomics. 2011 Feb 23;4:19. doi: 10.1186/1755-8794-4-19 (PMC3060843; doi:10.1186/1755-8794-4-19)
Supplement: Additional file 6 — This file contains the supplemental Tables referenced in this article. [file 1755-8794-4-19-S6.DOC]

**Supplementary Table Legends**

**Table 1.** This table shows the 15 miRNA with the highest fold change between RA and controls. In bold miRNAs that are not expressed in controls. The listed miRNAs are all up-regulated in RA.

**Table 2.** This table shows the 15 miRNA with the highest fold change between RAEB2 and controls. In bold miRNAs that are not expressed in controls. The listed miRNAs are all up-regulated in RAEB2. Note that the fold change for RA is even higher for a number of these miRNAs.

**Table 3.** This table shows the 15 miRNA with the highest fold change between RA and RAEB2. We underlined miRNAs that are not expressed in RAEB2. The listed miRNAs are all up-regulated in RA compared to RAEB2.

**Table 4** This table gives an overview on miRNAs and miRNA* that were detected only in the RA samples. We denoted known miRNA* with a single star and novel miRNA* with two stars.

**Table 5** This table shows folding information and read distributions for 10 novel miRNA*s detected in our experiments. The red base pairs correspond to the known mature sequence and the green nucleotides mark the principle location of aligned reads (the read with highest copy number among all samples was used). Note that due to RNase III activity, miRNA and miRNA* motifs, are often at locations that are not perfectly opposite (Zhang et al. 2010). In total reads on 29 miRNA* motifs had shifts of less than 3bp, nine had larger shifts and one sequence was expressed in very low copies.

**Supplementary Tables**

| miRNA Accession | Fold ChangeRA | Fold ChangeRAEB2 |
| --- | --- | --- |
| **mir-376a-2** | 3039.00 | N/A |
| **mir-376a-1** | 3039.00 | N/A |
| **mir-376c** | 2980.00 | N/A |
| **mir-144** | 2643.00 | 180.00 |
| **mir-374b*** | 1613.00 | N/A |
| **mir-374a*** | 1583.00 | N/A |
| **mir-126*** | 1253.00 | N/A |
| **mir-17** | 1176.00 | 92.00 |
| **mir-106a*** | 1176.00 | N/A |
| **mir-10a*** | 1134.00 | N/A |
| **mir-10b** | 1134.00 | 597.00 |
| **mir-20a** | 1110.00 | 92.00 |
| **mir-598**** | 733.00 | N/A |
| **mir-30d** | 699.00 | 87.00 |
| **mir-20b*** | 672.00 | N/A |
| Table 1 | | |

| miRNA Accession | Fold Change RA | Fold Change RAEB2 |
| --- | --- | --- |
| **mir-127** | 135.00 | 772.00 |
| **mir-10b** | 1134.00 | 597.00 |
| **mir-134** | 365.00 | 402.00 |
| **mir-155** | 452.00 | 271.00 |
| **mir-125b-2** | 87.00 | 264.00 |
| **mir-144** | 2643.00 | 180.00 |
| mir-1974** | 263.06 | 175.83 |
| **mir-1973**** | 34.00 | 112.00 |
| **mir-17** | 1176.00 | 92.00 |
| **mir-20a** | 1110.00 | 92.00 |
| **mir-139** | 169.00 | 90.00 |
| **mir-30d** | 699.00 | 87.00 |
| **mir-216b** | 27.00 | 76.00 |
| mir-19b-2* | 29.71 | 53.15 |
| mir-19b-1 | 29.71 | 53.07 |
| Table 2 | | |

| miRNA Accession | Fold ChangeRA|RABB2 |
| --- | --- |
| mir-451 | 981790 |
| mir-98** | 234378 |
| mir-29a* | 69761 |
| mir-29c* | 69229 |
| mir-29b-2* | 66760 |
| mir-29b-1* | 66760 |
| mir-16-1* | 61373 |
| mir-195* | 57990 |
| mir-191* | 43550 |
| mir-26a-2* | 34152 |
| mir-181b-2 | 30612 |
| mir-181b-1 | 30612 |
| mir-23b | 30357 |
| mir-101-1* | 24065 |
| mir-221* | 19347 |
| Table 3 | |

| **miRNA**   | **copy number** |  | **miRNA** | **copy number** |  | **miRNA** | **copy number** |  | | --- | --- | --- | --- | --- | --- | --- | --- | | mir-376a-1 | 3038 |  | mir-193b* | 147 |  | let-7g | 17 | | mir-376a-2 | 3038 |  | mir-1973 | 135 |  | mir-487a | 17 | | mir-376c | 2979 |  | mir-183* | 125 |  | mir-19b-2 | 16 | | mir-374b* | 1612 |  | mir-362** | 113 |  | mir-433 | 15 | | mir-374a* | 1582 |  | mir-1308** | 101 |  | mir-490 | 15 | | mir-126* | 1252 |  | mir-1977 | 99 |  | mir-1248 | 15 | | mir-106a* | 1175 |  | mir-499** | 99 |  | mir-29b-2 | 15 | | mir-10a* | 1133 |  | mir-887 | 88 |  | mir-629 | 14 | | mir-598** | 732 |  | mir-18b | 81 |  | mir-301b | 14 | | mir-20b* | 671 |  | mir-409 | 74 |  | mir-1974 | 13 | | mir-99b | 614 |  | mir-624 | 73 |  | mir-455 | 12 | | mir-22 | 512 |  | mir-1278 | 65 |  | mir-224 | 12 | | mir-503** | 452 |  | mir-495 | 64 |  | mir-138-1 | 11 | | mir-362 | 381 |  | mir-532 | 54 |  | mir-363 | 11 | | mir-497* | 322 |  | mir-628 | 54 |  | mir-138-2 | 11 | | mir-410 | 291 |  | mir-485 | 46 |  |  | | | mir-193b | 289 |  | mir-124-2 | 41 | | mir-140** | 285 |  | mir-124-3 | 41 | | mir-376b | 250 |  | mir-577 | 33 | | mir-15b* | 234 |  | mir-30c-2 | 32 | | mir-34a* | 229 |  | mir-505 | 31 | | mir-382 | 226 |  | mir-129-2 | 31 | | mir-582** | 212 |  | mir-766 | 27 | | mir-421** | 207 |  | mir-125a | 26 | | mir-551b | 206 |  | mir-200b | 26 | | mir-625 | 203 |  | mir-200c | 26 | | mir-188** | 201 |  | mir-219-2 | 25 | | mir-487b | 197 |  | mir-16-1 | 24 | | mir-1259** | 195 |  | mir-15a | 23 | | mir-16-2* | 185 |  | mir-944 | 20 | | mir-1290** | 175 |  | mir-326 | 20 | | mir-654 | 173 |  | mir-323 | 19 | | mir-320a** | 173 |  | mir-101-1 | 18 | | mir-590** | 152 |  | mir-450a-2 | 18 | |
| --- | --- | --- | --- | --- | --- | --- | --- | --- | --- | --- | --- | --- | --- | --- | --- | --- | --- | --- | --- | --- | --- | --- | --- | --- | --- | --- | --- | --- | --- | --- | --- | --- | --- | --- | --- | --- | --- | --- | --- | --- | --- | --- | --- | --- | --- | --- | --- | --- | --- | --- | --- | --- | --- | --- | --- | --- | --- | --- | --- | --- | --- | --- | --- | --- | --- | --- | --- | --- | --- | --- | --- | --- | --- | --- | --- | --- | --- | --- | --- | --- | --- | --- | --- | --- | --- | --- | --- | --- | --- | --- | --- | --- | --- | --- | --- | --- | --- | --- | --- | --- | --- | --- | --- | --- | --- | --- | --- | --- | --- | --- | --- | --- | --- | --- | --- | --- | --- | --- | --- | --- | --- | --- | --- | --- | --- | --- | --- | --- | --- | --- | --- | --- | --- | --- | --- | --- | --- | --- | --- | --- | --- | --- | --- | --- | --- | --- | --- | --- | --- | --- | --- | --- | --- | --- | --- | --- | --- | --- | --- | --- | --- | --- | --- | --- | --- | --- | --- | --- | --- | --- | --- | --- | --- | --- | --- | --- | --- | --- | --- | --- | --- | --- | --- | --- | --- | --- | --- | --- | --- | --- | --- | --- | --- | --- | --- | --- | --- | --- | --- | --- | --- | --- | --- | --- | --- | --- | --- | --- | --- | --- | --- | --- | --- | --- | --- | --- | --- | --- | --- | --- | --- | --- | --- | --- | --- | --- |
| Table 4 |

| mir-103-1** | 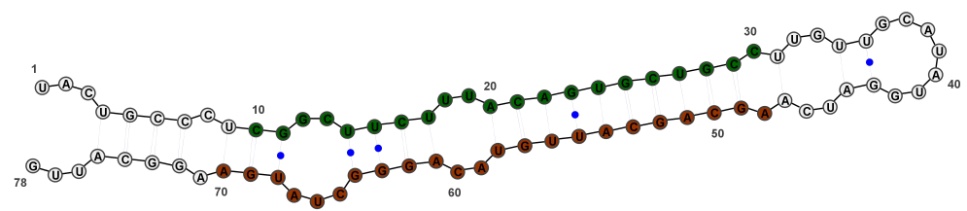 | 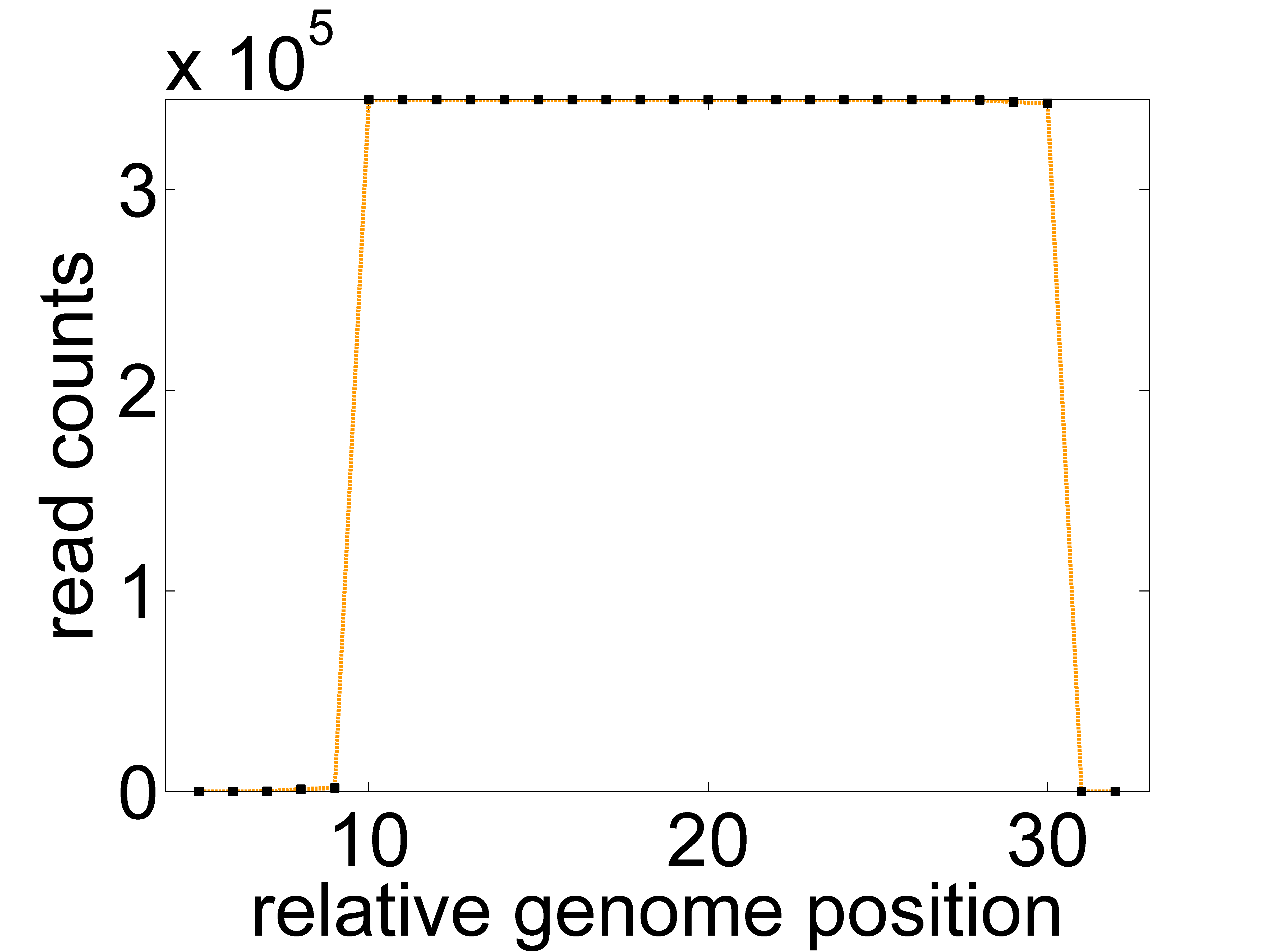 |
| --- | --- | --- |
| mir-107** | 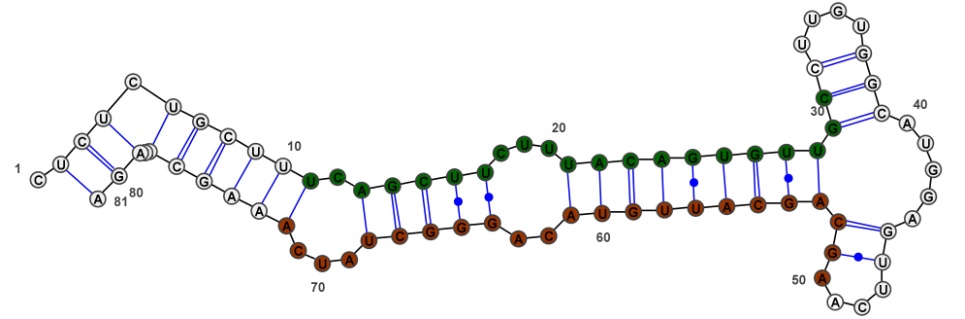 |
| mir-98** | 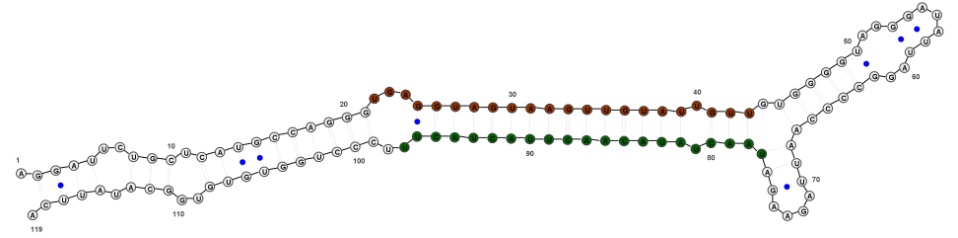 | 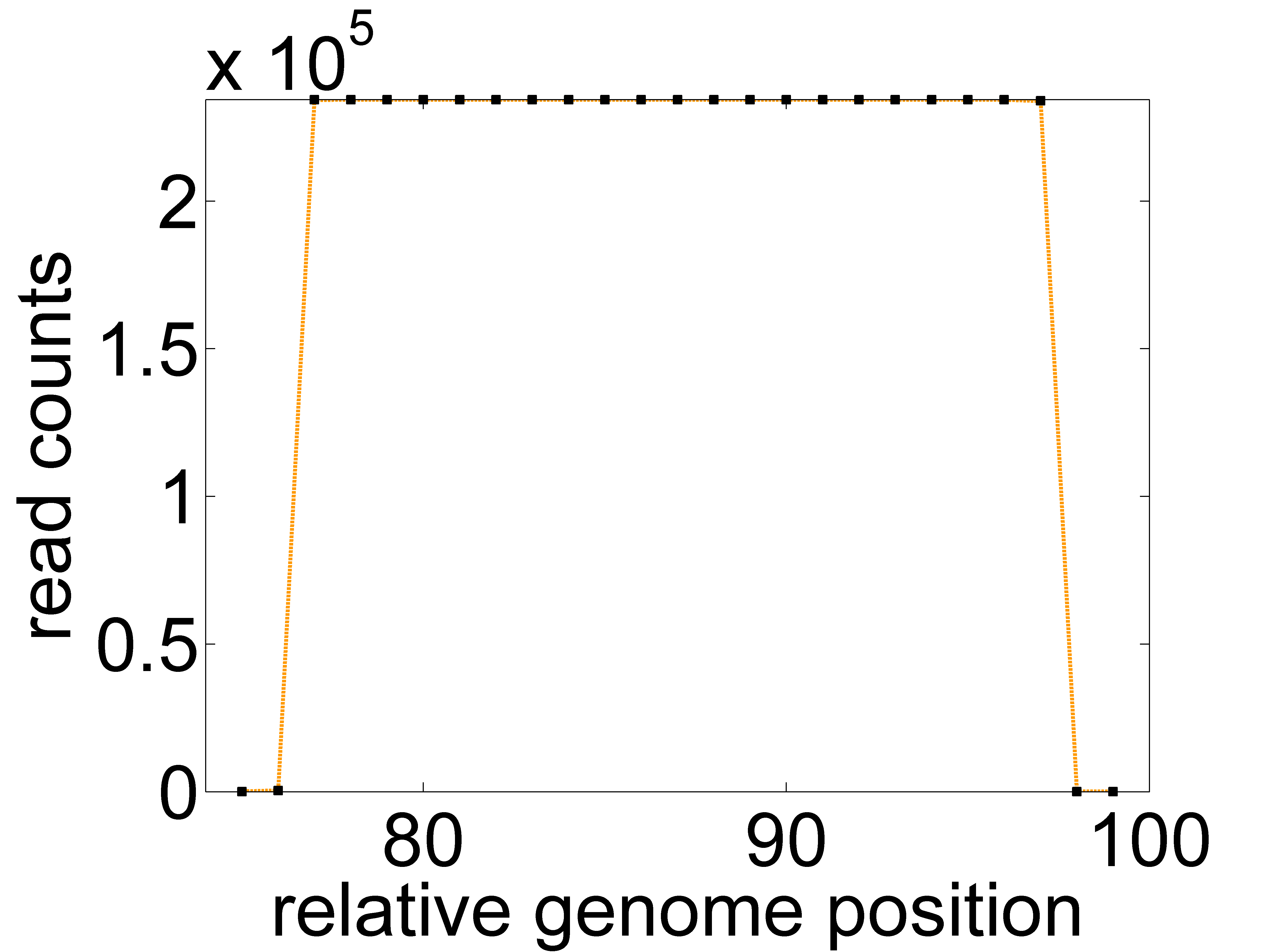 |
| mir-146b** | 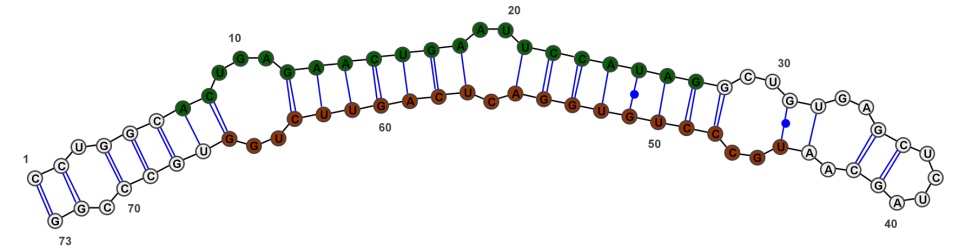 | 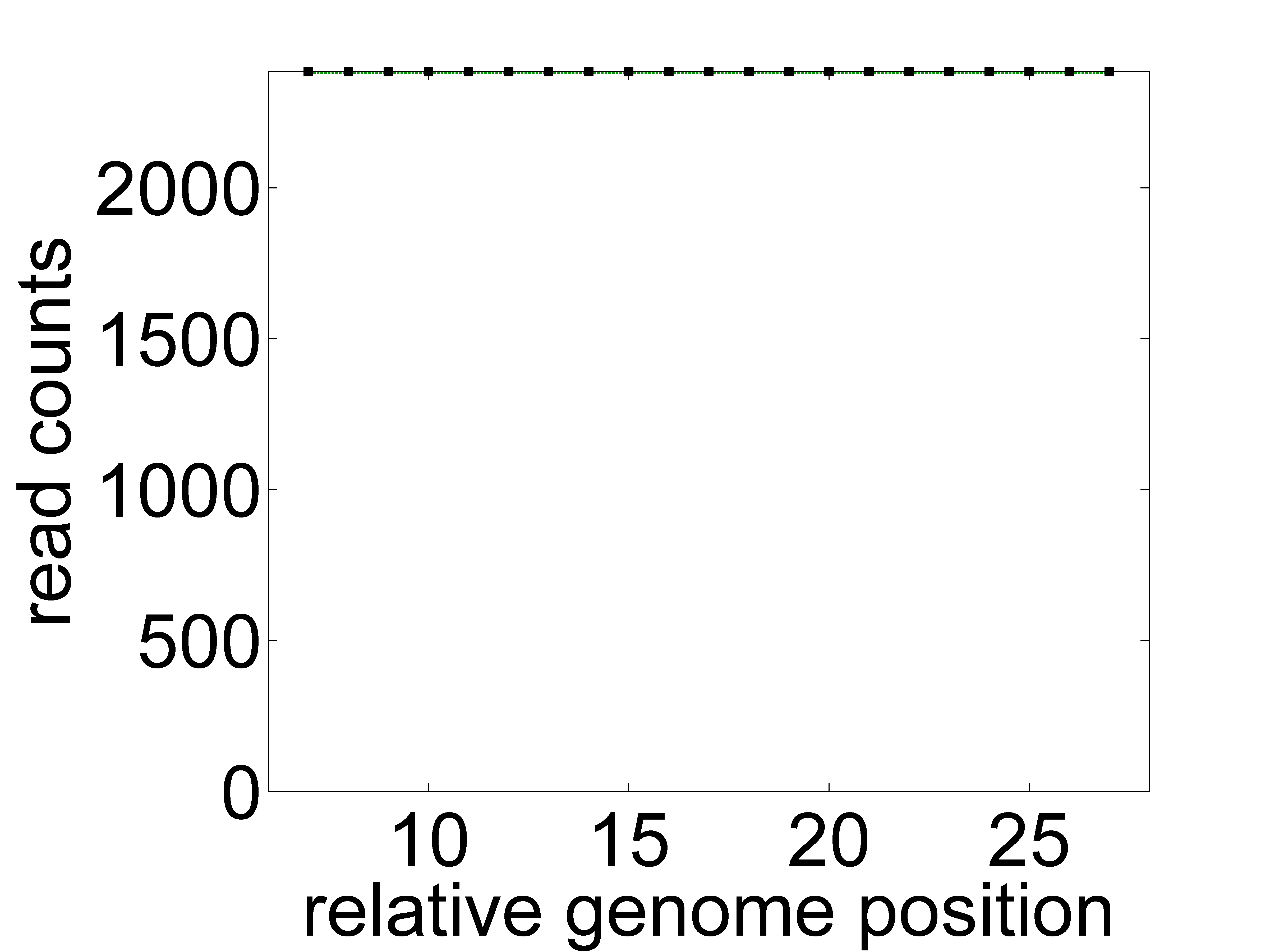 |
| mir-199a-1** | 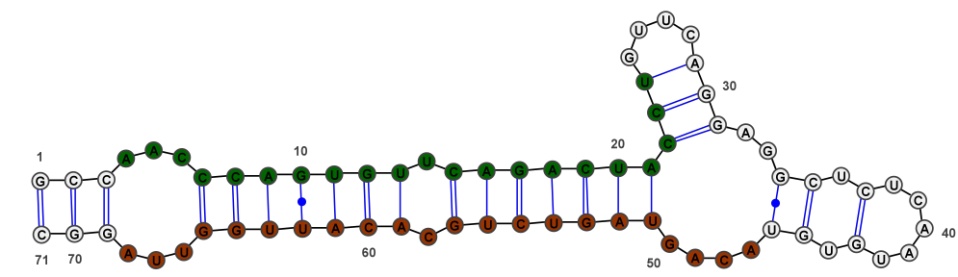 | 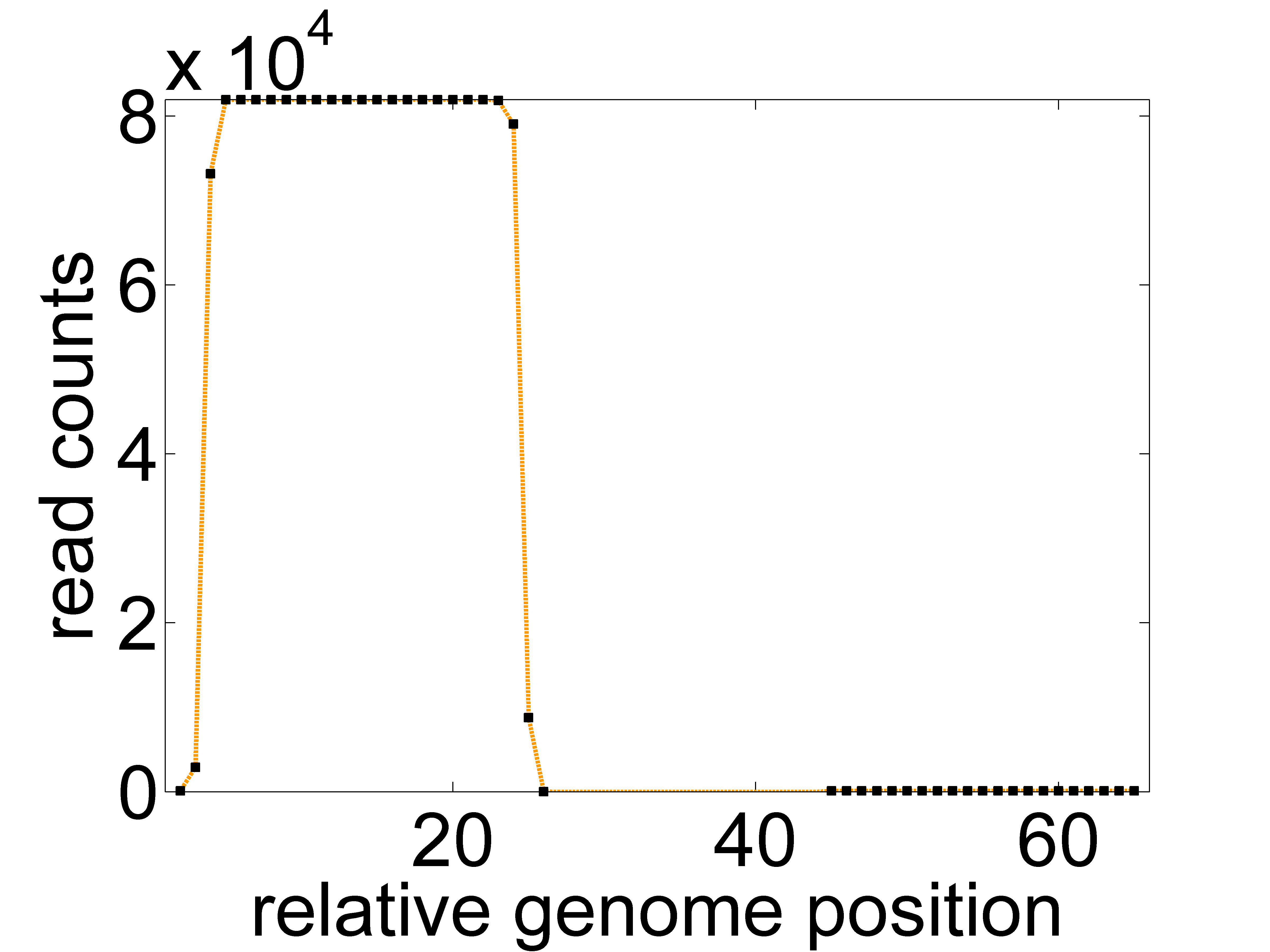 |
| mir-199b** | 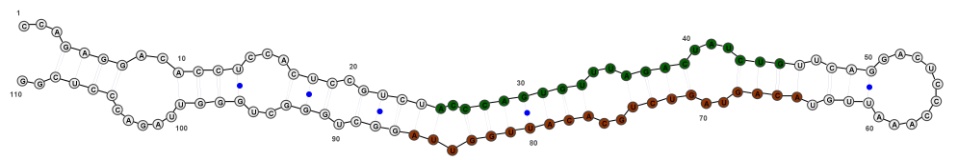 |
| mir-1974** | 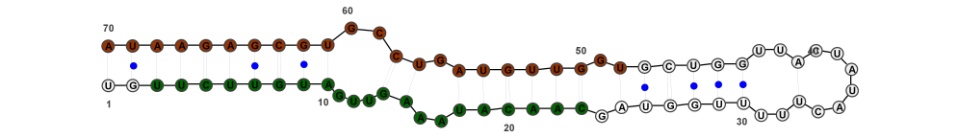 | 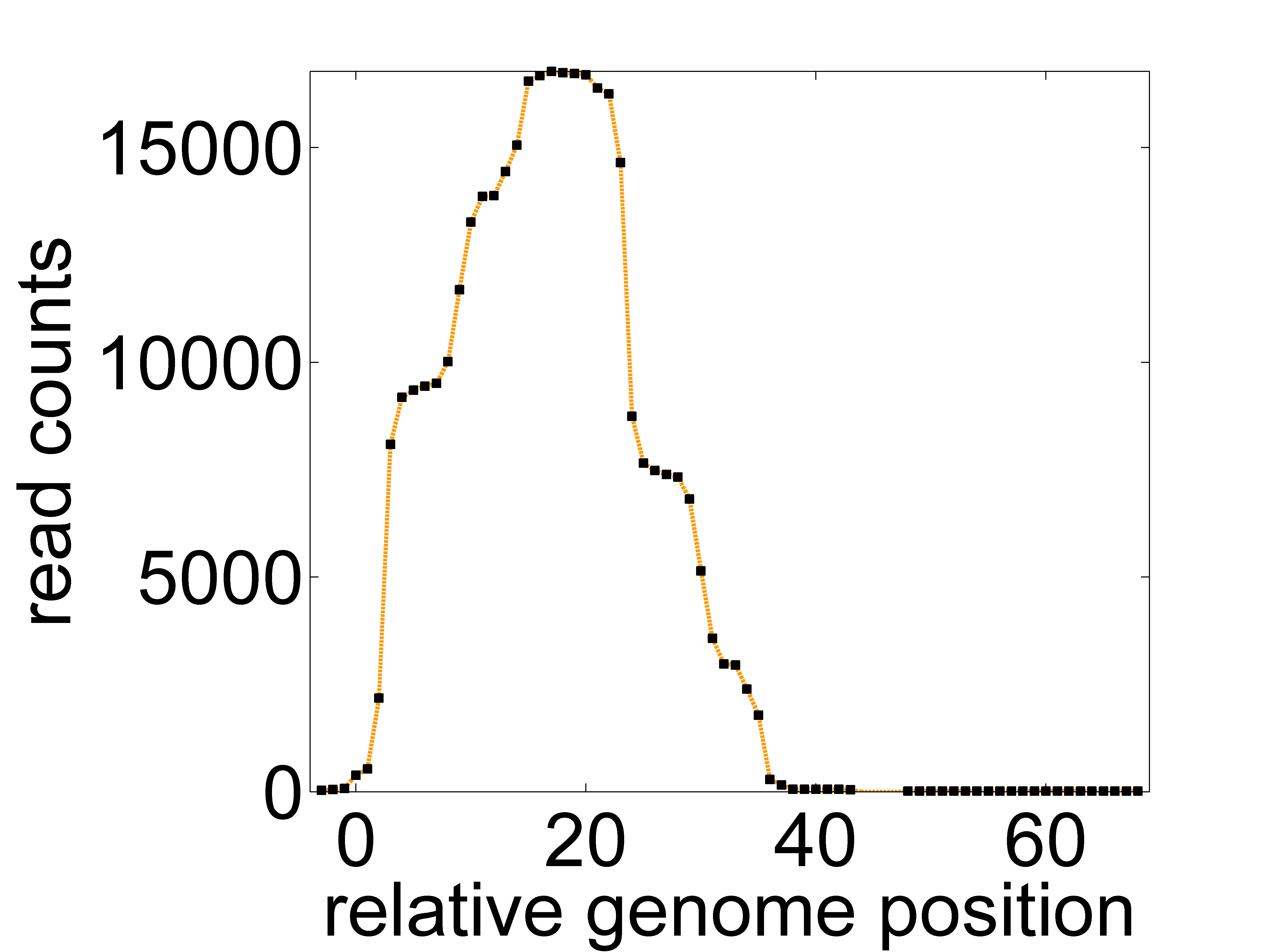 |
| mir-320d-1** | 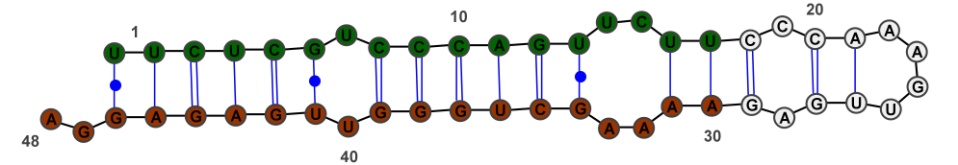 | 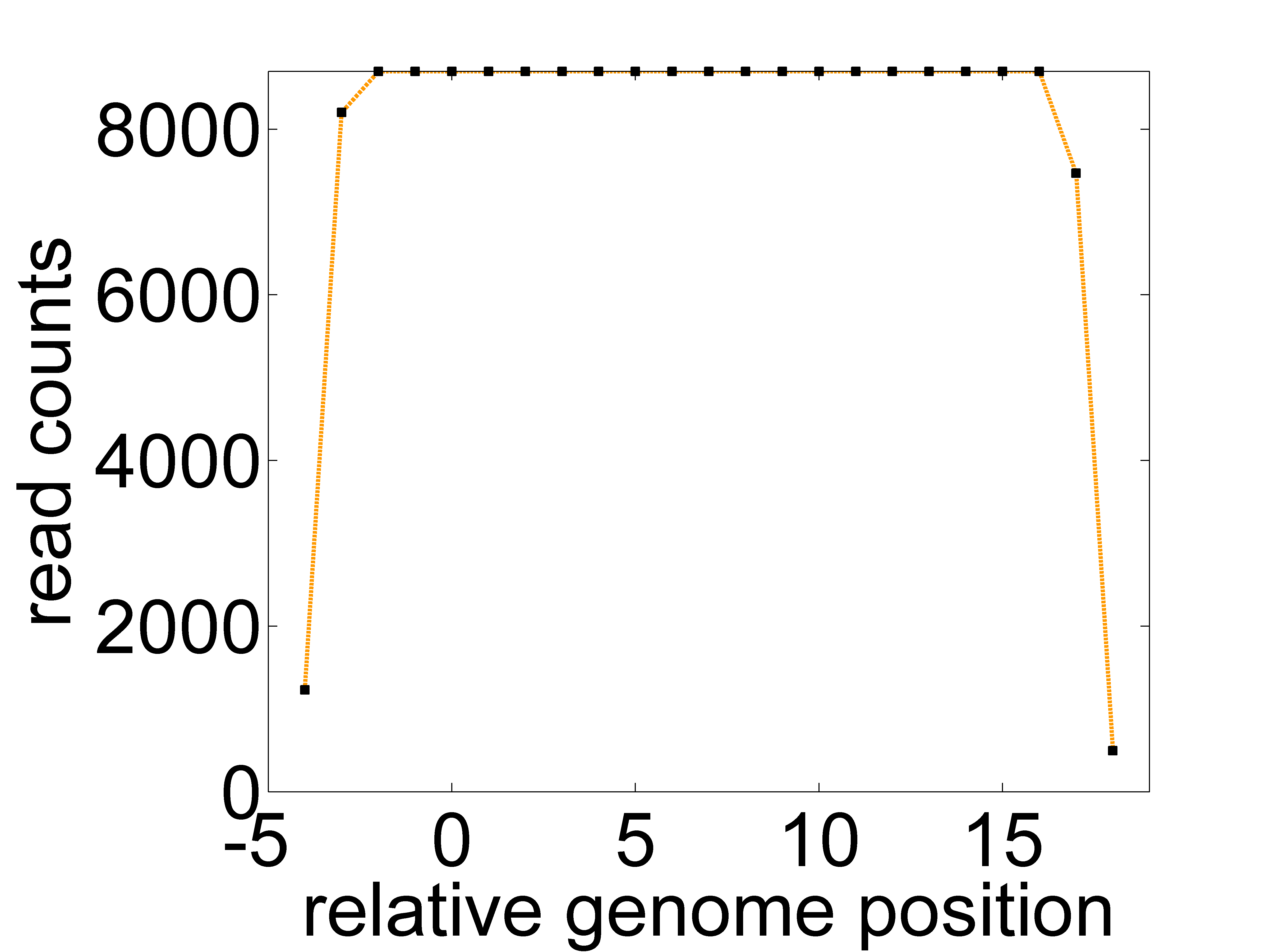 |
| mir-320d-2** | 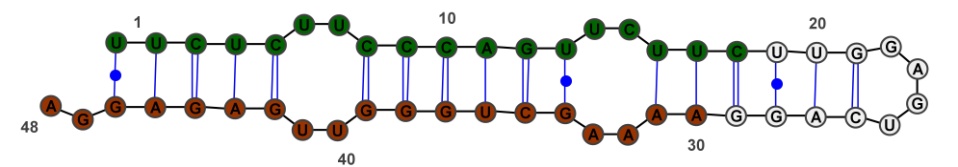 | 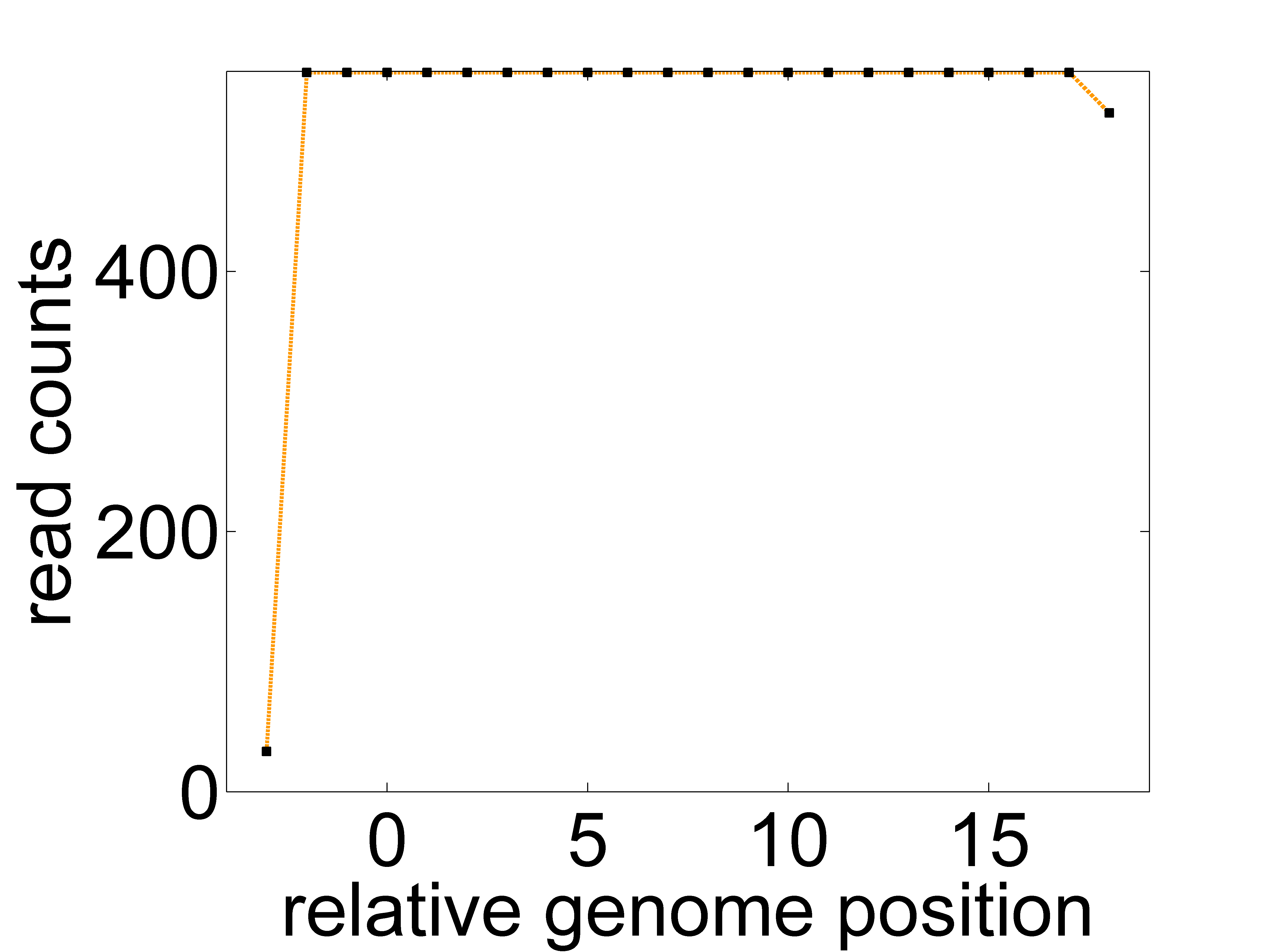 |
| mir-1282** | 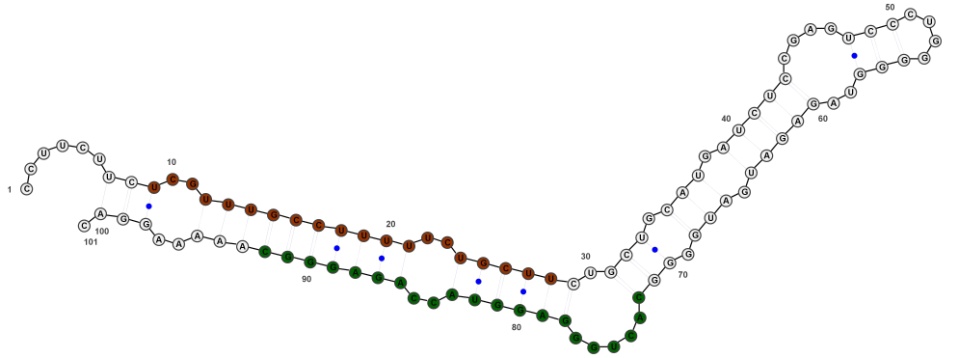 | 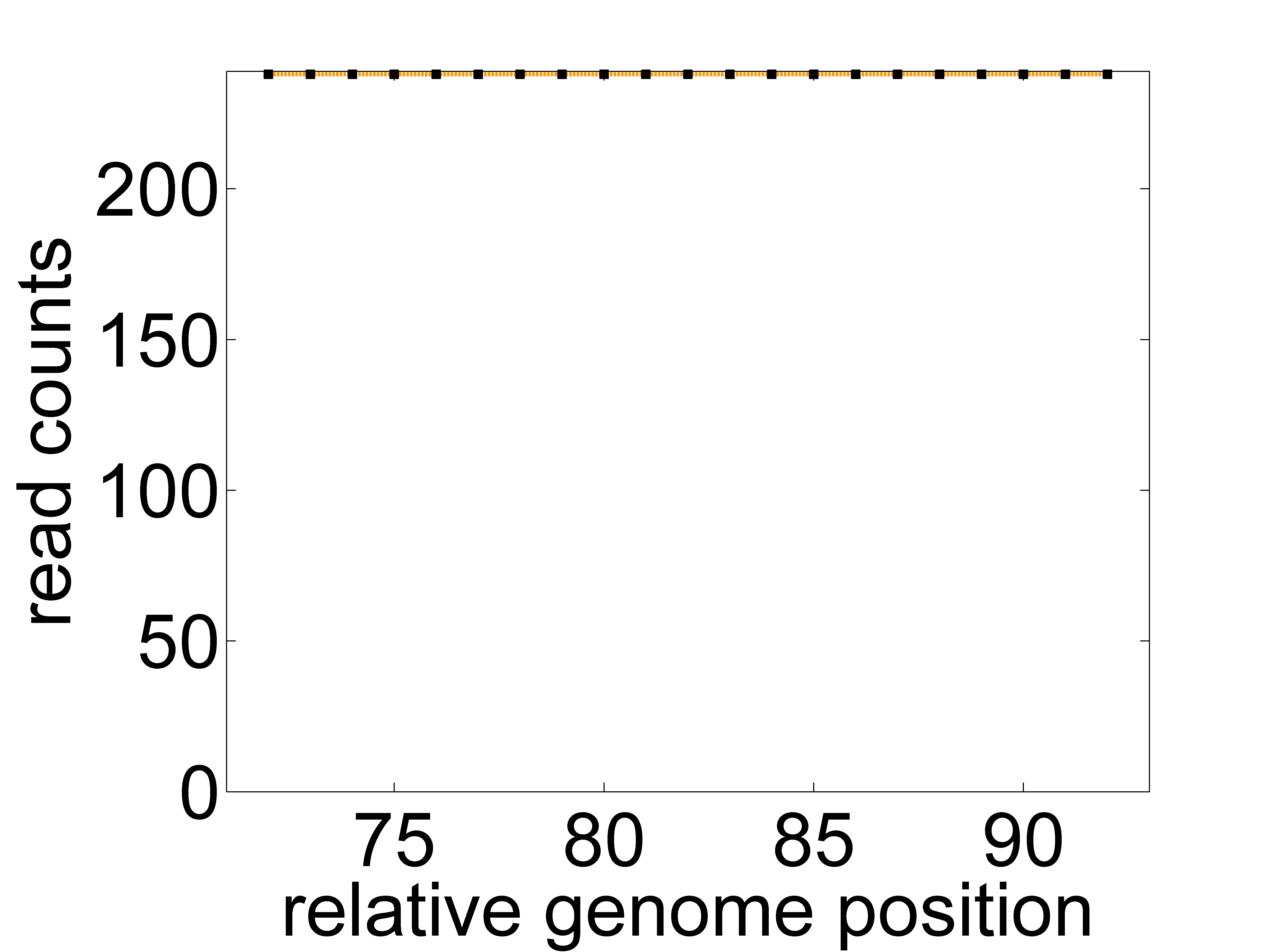 |
| mir-210** | 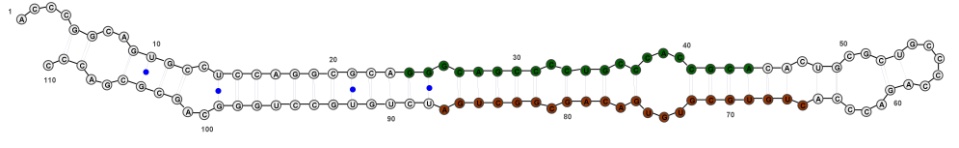 | 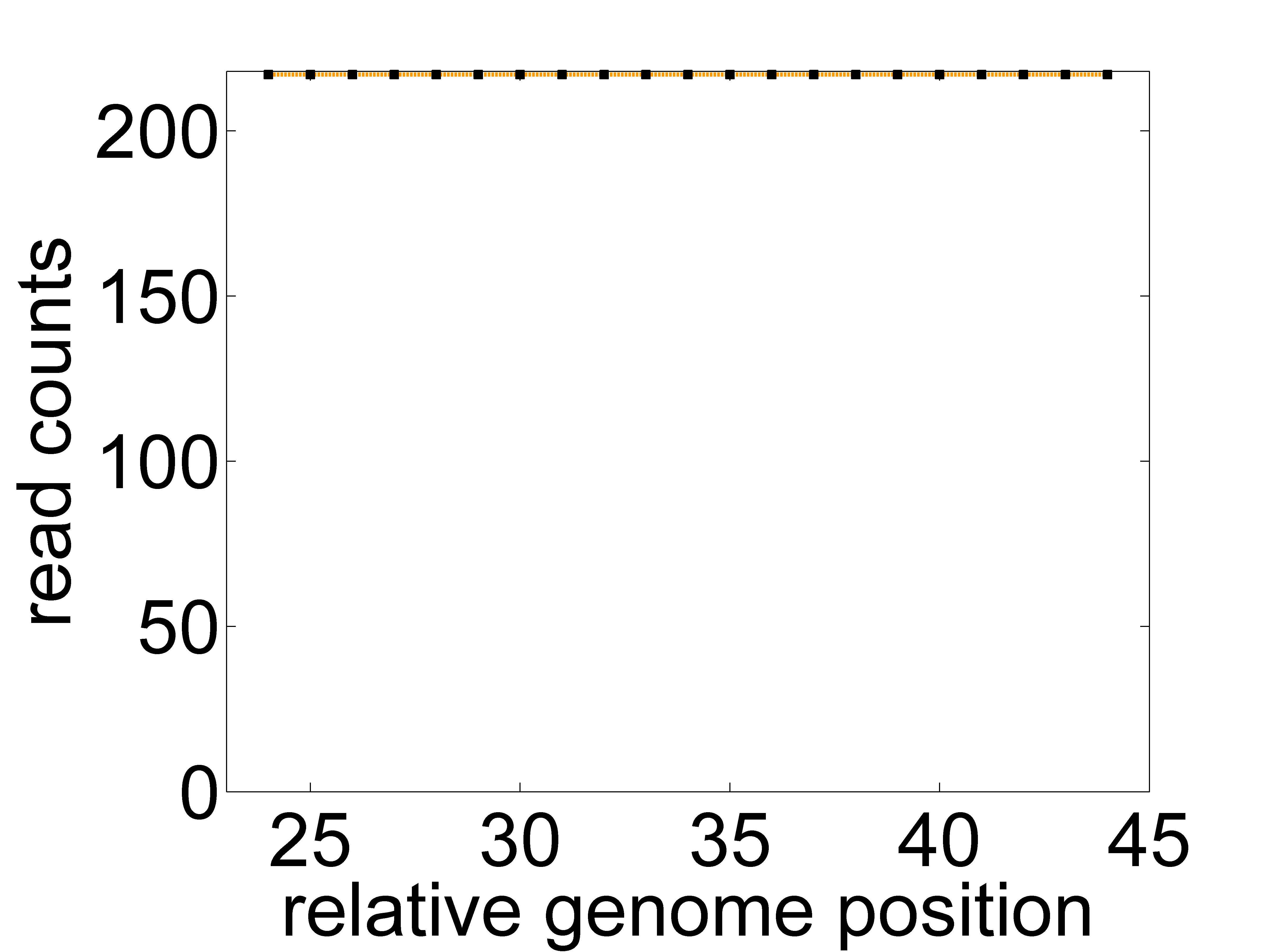 |
| mir-1246** | 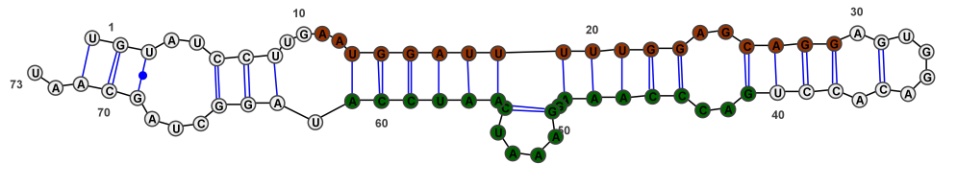 | 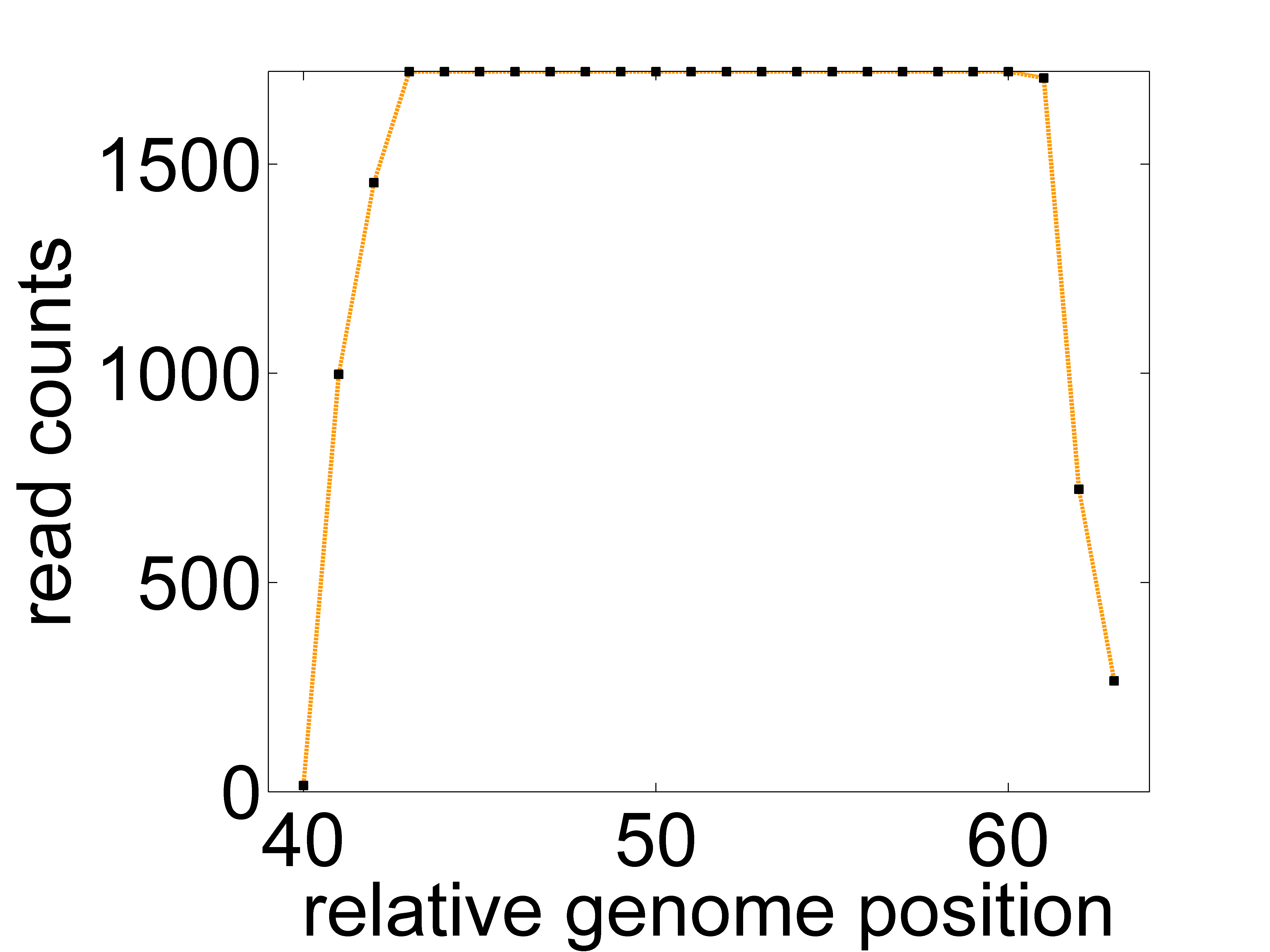 |
| Table 5 | | |

**References**

**Zhang W, Gao S, Zhou X, Xia J, Chellappan P, Zhang X, Jin H. 2010. Multiple distinct small RNAs originated from the same microRNA precursors. *Genome Biol* 11(8): R81.**
