# Supplementary material for: Integrative analysis of next generation sequencing for small non-coding RNAs and transcriptional regulation in Myelodysplastic Syndromes
Source: BMC Med Genomics. 2011 Feb 23;4:19. doi: 10.1186/1755-8794-4-19 (PMC3060843; doi:10.1186/1755-8794-4-19)
Supplement: Additional file 7 — This file contains the supplemental Text referenced in this article. [file 1755-8794-4-19-S7.DOC]

**Functional roles of miRNA and miRNA* in Myelodysplastic Syndromes:** Additional molecular functions identified for combined miRNA and miRNA* targets in RA and RAEB2

In our analysis, we further discovered that miRNA targets were enriched for the “Myc Mediated Apoptosis Signaling” pathway (RA: 3.02E-2) only in RA. Its central element, Myc, is known to amplify in cells transforming from MDS to AML (Milojkovic et al. 2005) and our exon array analysis showed a large fold change of its coding gene in RAEB2 (3.65) and RA(1.70).

Moreover, we found that genes involved in “RNA Damage and Repair” mechanisms are enriched miRNA targets only in RAEB2 (RAEB2: 3.59E-04). Similar to DNA, RNA damage can lead to apoptosis and cell cycle arrest. This goes along with enrichment for “RNA Post-Transcriptional Modification” (RA: 3.59E-04, RAEB2: 1.71-03) and “Post-Translational Modification” (RAEB2: 1.09E-02. RA:1.84E-03).

There was evidence that miRNAs interfere with a number of canonical pathways that are deregulated in low and high-grade MDS. In the low grade, pathways that reached significance include “Wnt/β-catenin Signaling”, notably one of the most down regulated pathways in MDS (Pellagatti et al. 2010), “TGF-β signaling” which can be inhibited to promote hematopoiesis in MDS (Zhou et al. 2008), as well as “IGF-1 Signaling” and, as mentioned above, “Myc Mediated Apoptosis Signaling”. In the high grade the “BMP signaling pathway” and “Cardiomyocyte Differentiation via BMP receptors”, which have been implicated in MDS (Andrieux et al. 2007), and the “Cell Cycle: G1/S Checkpoint Regulation”, were significant enriched for miRNA targets. We also found miRNA enrichment in RA and RAEB2 for physiological functions like “Hematological System Development and Function” and “Hematopoiesis” as well as for “Hematological Disease”, which further strengthened the relation of target genes with bone marrow or other blood forming organ disease.

Andrieux, J., Roche-Lestienne, C., Geffroy, S., Desterke, C., Grardel, N., Plantier, I., Selleslag, D., Demory, J.L., Lai, J.L., Leleu, X. et al. 2007. Bone morphogenetic protein antagonist gene NOG is involved in myeloproliferative disease associated with myelofibrosis. *Cancer Genet Cytogen* **178**(1): 11-16.

Milojkovic, D., Buggins, A.G., Devereux, S., Thomas, N.S., and Mufti, G.J. 2005. Tumor supernatant from myeloid malignancies inhibits T-cell apoptosis and cell cycle entry independently. *Leukemia* **19**(9): 1699-1702.

Pellagatti, A., Cazzola, M., Giagounidis, A., Perry, J., Malcovati, L., Della Porta, M.G., Jadersten, M., Killick, S., Verma, A., Norbury, C.J. et al. 2010. Deregulated gene expression pathways in myelodysplastic syndrome hematopoietic stem cells. *Leukemia* **24**(4): 756-764.

Zhou, L., Nguyen, A.N., Sohal, D., Ma, J.Y., Pahanish, P., Gundabolu, K., Hayman, J., Chubak, A., Mo, Y.K., Bhagat, T.D. et al. 2008. Inhibition of the TGF-beta receptor I kinase promotes hematopoiesis in MDS. *Blood* **112**(8): 3434-3443.
